# Bacterial extracellular vesicles promote membrane repair and tolerance to polymyxin B

**DOI:** 10.1126/sciadv.adx6378

**Published:** 2026-06-10

**Authors:** Julia Bos, Yasmina Abou Haydar, Olena Mayboroda, Anaïs Backland, Pierre-Henri Commere, Didier Mazel

**Affiliations:** ^1^ Institut Pasteur, Université Paris Cité, CNRS UMR3525, Unité Plasticité du Génome Bactérien, 75015 Paris, France.; ^2^ Sorbonne Université, Collège Doctoral, F-75005 Paris, France.; ^3^ Institut Pasteur, Université Paris Cité, Unité Biologie des Bactéries Intracellulaires, CNRS UMR6047, 75015, Paris, France.; ^4^ Institut Pasteur, Université Paris Cité, NanoImaging Core Facility, Paris, France.; ^5^ Unité Evo-Eco-Paléo, UMR 8198, Université de Lille, Lille, France.; ^6^ Institut Pasteur, Cytometry Platform, Université Paris Cité, 75015 Paris, France.

## Abstract

Bacterial extracellular vesicles (EVs) are nanosized lipid structures released under stress, yet their interactions with antibiotics remain poorly understood. We tracked real-time interactions between *Escherichia coli*, EVs, and fluorescent polymyxin B (Pmb) using single-cell imaging and cytometric approaches. EVs rapidly sequester Pmb, facilitating its removal from bacterial envelopes, and act as plugs by adhering to or fusing with damaged sites. Pmb triggers early Cpx/σE- and Rcs-dependent stress responses, linked to a ∼25% reduction in cell surface area, a ∼50-fold increase in vesiculation, and remodeling of membrane properties. After an adaptive lag phase, sustained EV release supports detoxification and envelope repair, enabling growth recovery and transient tolerance to Pmb. Together, these findings reveal previously unrecognized functions of EVs in membrane repair and tolerance to membrane-active antibiotics.

## Teaser

*E. coli* releases vesicles that repair membrane damage and promote survival under antibiotic stress.

## INTRODUCTION

The world is confronted with the rising threat of bacteria that are resistant to nearly all available antibiotics (*1*). Antimicrobial peptides (AMPs), such as polymyxins [i.e., polymyxin B (Pmb) and polymyxin E (colistin)], remain vital antibiotics of last resort due to their efficacy against multidrug-resistant (MDR) Gram-negative bacteria including critical pathogens (*2*), such as *Escherichia coli*, *Klebsiella* spp., and *Pseudomonas aeruginosa*, despite their reported nephrotoxicity (*3*, *4*). These cationic peptides, originally found in *Bacillus polymyxa* (*5*), are naturally produced by many organisms as part of their innate defense, as reviewed in (*6*).

The renewed use of polymyxins has prompted further investigation into their mechanisms of action and resistance, as reviewed in (*7*), which are linked to modifications in lipopolysaccharide (LPS) layer decorating the outer membrane of Gram-negative bacteria (*8*). These changes result in increased efflux, reduced porin permeability, and increased membrane blebbing (*9*). The identification of the mobile colistin resistance gene *mcr-1* (*10*) in *Enterobacteriaceae* isolates in China, highlighted the rise of horizontally acquired resistance genes, thereby impeding strategies for treating MDR infections.

Several models describe the interaction of polymyxins, notably Pmb, with bacterial membranes (*11*, *12*). Pmb initially binds to the negatively charged phosphate group of the lipid A core in the LPS of the outer membrane, neutralizing it (*13*). As the peptide progresses to the inner membrane, it disrupts the membrane structure, causing leakage and cell death. The membrane-disrupting effects of Pmb trigger several conserved membrane stress responses (*14*) that enable bacteria to adapt to the antibiotic stress as reviewed in (*7*, *15*). In bacteria and particularly in *E. coli*, the Cpx (envelope stress response), Rcs (regulator of capsule synthesis), and σE [the alternative sigma factor (RpoE)] systems are activated in response to Pmb treatment (*16*–*18*). The σE pathway and Cpx two-component system (*15*, *19*) manage misfolded proteins and repair membrane damage, while the Rcs two-component pathway, regulated by RcsC, RcsA, and RcsB, responds to envelope stress affecting the outer membrane and peptidoglycan layer (*15*, *20*–*23*). In addition, some bacteria use PhoP/PhoQ (*24*) and PmrA/PmrB (*25*, *26*) two-component regulatory systems to modify their outer membrane under polymyxin exposure.

Beyond these endogenous mechanisms, extracellular vesicles (EVs) have emerged as key mediators involved in the development of antimicrobial resistance, as reviewed in (*27*). These nanosized lipid-enclosed particles, released in response to stress, sequester antibiotics and protect bacterial populations by reducing local antibiotic concentrations and shielding cell membranes (*27*). In particular, EVs have been implicated in polymyxin resistance (*28*–*34*) in microorganisms such as *E. coli* (*28*, *35*), *Acinetobacter baumannii* (*31*), *Pseudomonas syringae* (*30*), *Salmonella enterica* serovar Typhi (*33*, *34*), *and Pseudomonas aeruginosa PAO1* (*32*) in which EV production alleviates immediate antibiotic stress by sequestering the Pmb, colistin, or melittin drugs. In particular, EVs purified from Pmb-resistant strains protect MDR strains against the bactericidal effect of Pmb (*28*, *31*), suggesting that EVs extend the spectrum of drug resistance.

Despite growing evidence that vesiculation constitutes an intrinsic bacterial stress response (*36*, *37*) linked to antibiotic tolerance, critical questions remain regarding whether and how EVs directly interact with bacterial membranes. Current models emphasize EV-mediated drug sequestration (*28*, *29*, *31*), but the ability of bacterial membranes to take up EVs remains uncertain, and the potential advantages this might confer are likewise not established. Unlike eukaryotic cells, where protein complexes mediate vesicle fusion (*38*–*41*), the analogous processes in bacteria are not known. Some studies suggest that ESCRT-like proteins may play a role in bacterial EV dynamics (*42*), but their function in vesicle formation, release, or membrane fusion remains speculative, and more research is needed to capture fusion events and to identify any potential fusion machinery.

Here, we investigate the real-time interplay between EVs, Pmb, and *E. coli* under subinhibitory envelope stress by Pmb. By combining high-resolution fluorescence microscopy, flow cytometry, cryo–electron microscopy (cryo-EM), and single-cell analyses, we identify EVs as key players in bacterial membrane adaptation. We show that beyond a decoy function, EVs remove and/or patch damaged regions through adhesion or fusion to stressed cells, linking vesiculation to membrane repair and growth recovery. Pmb stress triggers an early Rcs- and Cpx/σE-dependent envelope stress response associated with morphological remodeling and increased EV production, while vesiculation persists beyond stress signaling to support adaptation. Together, these findings position EV production and uptake as coordinated processes that promote membrane protection and transient tolerance.

## RESULTS

### Adaptive responses of *E. coli* to Pmb exposure

**Fig. 1.**
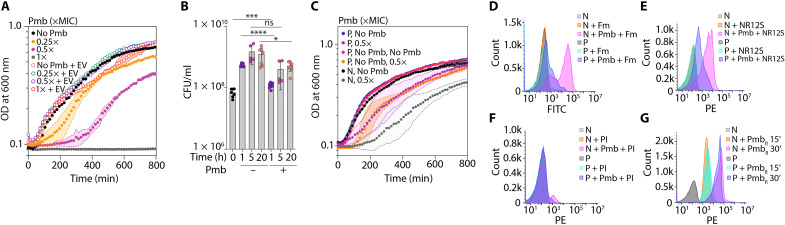
Growth dynamics and adaptive responses of *E. coli* following Pmb stress. (**A**) Growth curves of WT bacteria in the absence or presence of various doses of Pmb (0.25×, 0.5×, and 1× MIC; filled/plain circles) with or without the concomitant addition of pure EVs (empty circles). *n* = 3. (**B**) Survival assays of WT cells grown in the presence or absence of Pmb (0.5× MIC). Viable cells were quantified by CFU counting at 1, 5, and 20 hours (h) after antibiotic addition (*n* = 5 to 9 biological replicates). Statistical significance was assessed [Welch’s *t* test; not significant (ns), *P* = 0.057; *P = 0.04; ***P = 0.0004; ****P < 0.0001]. (**C**) Growth curves of naïve (“N”) and Pmb-adapted cells (progeny, “P”) in the presence or absence of Pmb (0.5× MIC). Naïve cells (black/dark gray circles) were never exposed to Pmb, whereas adapted cells (colored circles) were previously grown at 0.5× MIC Pmb. Cells were passaged with or without Pmb and, when indicated, subsequently reexposed under identical conditions. Changes in lag phase duration were used as a proxy to evaluate adaptive growth responses in progeny cells after passage with or without Pmb (*n* = 3). In all plots, error bars represent SD. (**D** to **G**) Representative single-cell flow cytometry plot showing membrane properties of Pmb-adapted progeny and naïve cells. Data show means ± SD (*n* = 3; except for PI, *n* = 2; 50,000 events per sample). Membrane permeability (D) was assessed using Fm1-43 [fluorescein isothiocyanate (FITC) channel], membrane fluidity (E) using the solvatochromic probe NR12S, monitored by a shift toward red emission [phycoerythrin (PE) channel], and membrane integrity (F) using propidium iodide (PI) (PE channel). Binding of fluorescently labeled Pmb (Pmb_fl_) (PE channel) was also assessed (G).

We investigated the adaptive responses of live *E. coli* exposed to sub–minimum inhibitory concentration (sub-MIC) levels of Pmb, a membrane-active antibiotic. Wild-type (WT) cells grown in LB resumed growth after exposure to 0.25× and 0.5× MIC Pmb, recovering after lag phases of ∼150 and ∼360 min, respectively (Fig. 1A). During these lag phases, cells experienced transient growth arrest at 1 hour (∼1.7-fold increase compared with ∼8-fold in untreated controls) and reduced growth (5 hours) (∼5.6-fold versus ∼21-fold) but did not die, as confirmed by colony-forming unit (CFU) assays when populations are normalized to their initial CFU counts (time 0) (Fig. 1B). In contrast, cultures treated with 1× MIC Pmb failed to resume growth (Fig. 1A). These results indicate that WT bacteria can rapidly adapt to subinhibitory Pmb exposure.

To determine whether this recovery arises from genetic resistance or physiological tolerance, we performed whole-genome sequencing on the EV-exposed, Pmb-adapted populations. No mutations were detected (see Materials and Methods), indicating that the response represents tolerance rather than heritable resistance. Progeny derived from Pmb-adapted cells, capable of growing after prolonged exposure to 0.5× MIC Pmb but still susceptible to lethal Pmb concentrations (Fig. 1C), no longer exhibited the extended lag phase characteristic of their parents (naïve cells). However, this growth advantage was progressively lost when cells were passaged in the absence of Pmb, demonstrating that the adaptation is reversible and maintained only under continued antibiotic stress (Fig. 1C). A similar but weaker adaptive response was observed after exposure to 0.25× MIC Pmb (fig. S1A), consistent with a graded stress response.

We next examined membrane properties in adapted progeny compared with naïve cells. We performed single-cell fluorescence analyses, using both microscopy and flow cytometry, with membrane-specific dyes: Fm1-43 to probe permeability, propidium iodide (PI) to detect loss of membrane integrity, and the solvatochromic NR12S probe to monitor membrane fluidity (Fig. 1, D to G, and fig. S1, B and C). After sub-MIC exposure (0.5× MIC, 1 hour), adapted progeny showed minimal differences in Fm1-43 staining relative to naïve cells (26 ± 7% versus 73 ± 25%; Fig. 1D), displayed fewer PI-positive cells (2.0 ± 0.6% versus 7.5 ± 3.6%; Fig. 1E), and reduced NR12S red shift signals relative to naïve cells (48 ± 17% versus 70 ± 5%; Fig. 1F). Reduced NR12S red shift is consistent with increased lipid order, as evidenced by higher lipid order ratios (LORs) in adapted progeny compared with naïve cells exposed to Pmb (0.36 versus −0.92; fig. S1C and see Materials and Methods). These results demonstrate that Pmb-adapted progeny exhibit broad remodeling of membrane physical properties characterized by increased membrane order relative to posttreatment cells.

Despite these changes, adapted cells bound Rhodamine B–conjugated Pmb [fluorescently labeled Pmb (Pmb_fl_)] with similar efficiency to naïve cells and exhibited comparable Pmb_fl_ insertion ability after 30 min of treatment (Fig. 1G) and decay kinetics of membrane-associated Pmb_fl_ as shown by the Tau (τ) values (time constant; τ = 136.8 min versus 135.6 min; fig. S1, D and E). This suggests that adaptation does not reduce the interaction between Pmb and the membrane. Instead, tolerance likely arises downstream of binding, through altered membrane organization. Thus, the recovery of progeny cells reflects a reversible, stress-induced tolerance.

### Immediate cellular response to Pmb exposure

**Fig. 2.**
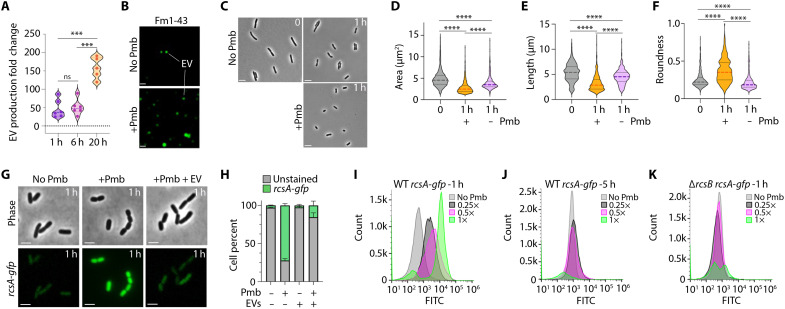
Rapid envelope stress responses and EV release upon Pmb exposure. (**A**) Quantification of EV production over time following growth with Pmb (0.5× MIC) using nano–flow cytometry. EV levels are presented as fold change relative to the untreated condition (*t* = 0) at 1 hour (*n* = 6), 6 hours (*n* = 6), and 20 hours (*n* = 5). Statistical significance was assessed (Welch’s *t* test; ns, *P* = 0.72; ***P = 0.0002). (**B**) Representative fluorescence microscopy images of purified EVs in the presence or absence of Pmb. EVs were immobilized on agarose pads containing the lipophilic dye Fm1-43 (green) and appear as bright fluorescent foci. Scale bars, 1 μm. (**C**) Representative phase contrast images of WT bacteria in mid-exponential phase (*t* = 0) and after 1 hour of growth with or without Pmb, illustrating morphological changes associated with envelope stress. (**D** to **F**) Quantification of cell area (D), length (E), and roundness (F) derived from the images in (C) using FIJI. Measurements performed on *n* = 629 (*t* = 0), *n* = 428 (1 hour without Pmb), and *n* = 938 cells (1 hour with Pmb). Values of mean cell area (2D), mean cell length, and mean roundness are indicated in table S3. Mean and quartiles are indicated (dashed lines). Statistical significance was assessed (Welch’s *t* test; ****P < 0.0001). (**G**) Microscopy snapshots of WT cells (*rcsA-gfp*) exposed to Pmb (0.5× MIC, 1 hour), with or without purified EVs, corresponding to the quantification in (H). Phase contrast and FITC (*rcsA-gfp*) images are shown. Scale bars, 2 μm. (**H**) Single-cell quantification showing the percentage of cells expressing *rcsA-gfp*, with or without EVs, at 60 min after Pmb addition. (**I** to **K**) *rcsA-gfp* expression analyzed by flow cytometry at time 1 hour (I) and 5 hours (J) after Pmb addition in WT cells and at 1 hour after Pmb addition in Δ*rcsB* cells (K). For (H) to (K), data show means ± SD (*n* = 3; 50,000 events per sample).

To examine how the response of naïve *E. coli* to Pmb unfolds over time, we monitored membrane remodeling, its redistribution, and envelope stress reporter activity. First, we quantified EV production during the onset of membrane stress. Sub-MIC Pmb triggered a rapid and sustained increase in vesiculation: EV output rose within the first hour by ∼50-fold, continued to accumulate through the lag phase, and reached 84 ± 26–fold increase by 20 hours (Fig. 2, A and B). EV levels at 6 hours (1.5 × 10^10^ ± 5.7 × 10^9^ EVs/ml) coincided with the resumption of growth (Fig. 1A), suggesting that high vesiculation supports recovery from membrane damage and enable cells to resume proliferation under sub-MIC Pmb.

Microscopy-based cell morphology measurements showed that morphological changes were associated with this early burst of vesiculation. During the first hour of Pmb exposure, cells became shorter and rounder, exhibiting a significant decrease in mean two-dimensional (2D) surface area from 3.97 to 2.91 μm^2^ (Fig. 2, C to F, and table S3). Using CFU-based cell counts combined with both projected 2D areas and a 3D-corrected spherocylinder model, we estimated that this morphological change corresponds to a ∼26% reduction in per-cell membrane surface area during the first hour of treatment (table S3).

The Rcs system responds to a range of envelope perturbations, including β-lactam antibiotics (*22*), lysozyme (*43*), changes in membrane lipid composition (*44*), and AMPs (*17*, *45*) such as Pmb. We thus monitored Rcs pathway activation using an *rcsA-gfp* reporter as a readout of envelope stress. We observed rapid, strong, and dose-dependent induction of *rcsA-gfp*: ∼65 ± 14% of cells responded within 1 hour (Fig. 2, G to I), showing three-, five-, and ninefold increases at 0.25×, 0.5×, and 1× MIC (fig. S2A), respectively. Additional reporters fused to *gfp*, such as *osmB* (an RcsB target) (*22*) and *htrA* also known as *degP* (a Cpx/σE-responsive protease) (*19*), were also activated by Pmb (fig. S2, B and C), consistent with simultaneous activation of both the Rcs and Cpx/σE envelope-stress pathways. However, both *osmB* and *htrA* showed a less strong induction in response to 0.5× Pmb compared to *rcsA* (fig. S2, E and F).

To distinguish early versus sustained stress signaling, we quantified *rcsA-gfp* expression at 1 and 5 hours after Pmb exposure (Fig. 2, I and J, and fig. S2, D to I). Expression of *rcsA-gfp* dropped sharply by 5 hours across all Pmb concentrations (Fig. 2J and fig. S2I). A similar trend was observed for *osmB* and *htrA* expression (fig. S2, G to I), supporting exit from the acute stress state. Time-lapse microscopy confirmed that *rcsA* expression decreased gradually during the lag phase and was substantially reduced by the end of the experiment (fig. S2J), concomitant with EV accumulation (Fig. 2A). As cells recovered, population-level *rcsA-gfp* approached basal levels (fig. S2A), revealing a temporal correlation between growth recovery, attenuation of membrane stress signaling, and increased EV levels.

Consistently, Δ*rcsB* cells failed to rapidly induce *rcsA-gfp* (Fig. 2K and fig. S3A) and displayed altered Pmb sensitivity: reduced sensitivity at low Pmb doses (0.25× MIC; fig. S3B) but increased sensitivity at moderate doses (0.7× MIC; fig. S3C). We also found that RcsB influenced baseline vesiculation: Δ*rcsB* cells vesiculated more under unstressed conditions, whereas Pmb exposure induced EV production to WT levels (fig. S3D), indicating that Pmb-triggered vesiculation occurs independently of Rcs signaling. These results showed that while RcsB modulates *rcsA* expression during the adaptive response to sub-MIC membrane stress, it is not strictly required for survival and vesiculation.

To test the contribution of EVs to stress attenuation, we supplemented cultures with purified EVs at near-physiological concentrations (8 to 160 EVs per cell; table S2). EV addition supported growth under Pmb (Fig. 1A) without activation of stress reporter *rcsA-gfp* (fig. S2A). This protective effect was specific to membrane-targeting antibiotics: EVs rescued cells exposed to Pmb or colistin even at high concentrations (fig. S4A) but had minimal impact on tobramycin or ciprofloxacin (fig. S4, B and C). Heat-treated EVs retained a protective activity (fig. S5A), indicating that membrane lipids, rather than intact vesicles or proteins, are sufficient for protection. EV-mediated rescue seemed to be independent of vesicle origin, as EVs from different strain backgrounds restored growth to a similar extent when normalized for concentration (figs. S3, B and C, and S5, B to D). Together, these results reveal a coordinated early response to Pmb: rapid vesiculation, cell compaction, activation of envelope stress pathways that returns to basal activity as EVs accumulate and growth resumes.

### Pmb_fl_ efficiently binds to cell membranes and triggers RcsA-dependent envelop stress response

**Fig. 3.**
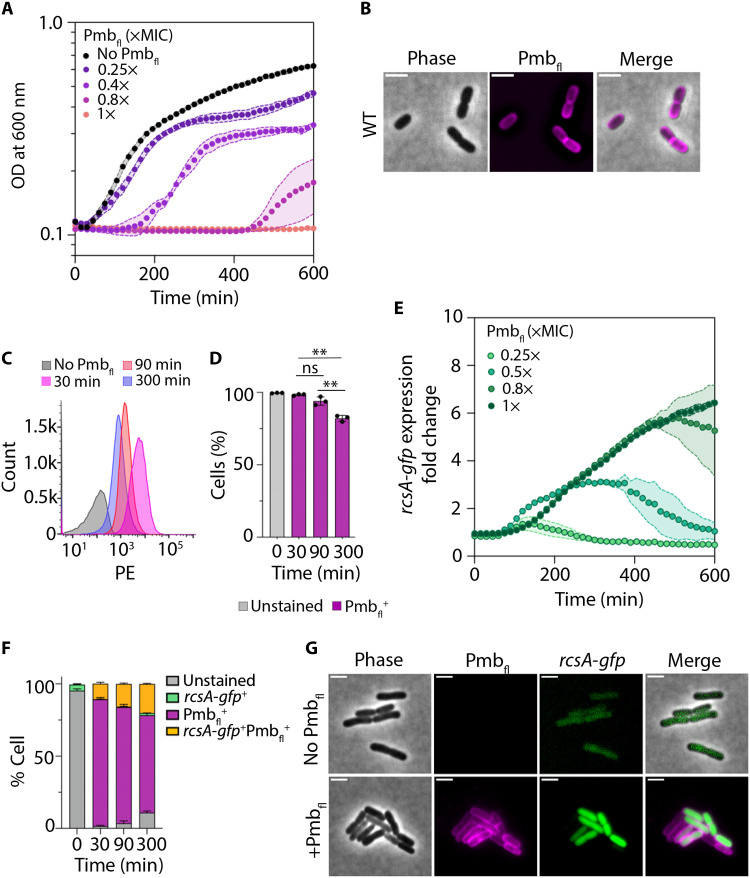
Pmb_fl_ fluorescent antibiotic decorates *E. coli* outer membranes and triggers membrane stress response. (**A**) Growth curves of WT bacteria in the absence or presence of various concentrations of Pmb_fl_. Data show means ± SD (*n* = 3). (**B**) Microscopy images showing Pmb_fl_ insertion (after 30 min) into cell membranes. Phase contrast, tetramethyl rhodamine isothiocyanate (TRITC; red; Pmb_fl_^+^), and merged images are displayed. Scale bar, 2 µm. (**C**) Representative analysis of Pmb_fl_ binding in *rcsA-gfp* bacteria by single-cell flow cytometry (PE channel) at 0, 30, 60, and 300 min following addition of Pmb_fl_ at 0.5× MIC. Data show means ± SD (*n* = 3; 50,000 events per sample). (**D**) Single-cell quantification of the Pmb_fl_ binding time course shown in (C), as measured by flow cytometry. Statistical significance was assessed [Welch’s *t* test; ns, *P* = 0.11 (30 to 90 min); **P = 0.001 (30 to 300 min); **P = 0.004 (90 to 300 min)]. (**E**) Expression levels of membrane stress response over time, as a function of various Pmb_fl_ concentrations. Expression fold change varies from 3× (0.4× Pmb_fl_) to 7× (1× Pmb_fl_). Data show means ± SD (*n* = 3). (**F**) Single-cell quantification plot (flow cytometry) showing the percentage of cells expressing *rcsA-gfp*^+^ (green), decorated with Pmbfl^+^ (purple), *rcsA-gfp*^+^–stressed cells decorated with Pmbfl^+^ (orange), and not fluorescent (unstained) cells across various exposure times to 0.5× MIC Pmb_fl_ or in the absence of Pmb_fl_. Data show means ± SD (*n* = 3). (**G**) Microscopy images of *rcsA-gfp* cells cultured to exponential phase and treated with or without 0.5× MIC Pmb_fl_ for 30 min. Phase contrast, fluorescent images in the TRITC channel (Pmb_fl_), FITC channel (*rcsA-gfp*), and merged images are shown. Scale bar, 2 µm.

To gain knowledge on the mechanisms underlying Pmb tolerance at the single-cell level, we studied the interaction between the antibiotic, the EVs, and the bacteria using Pmb_fl_, a derivative of Pmb conjugated to the fluorescent dye Rhodamine B. We determined that the MIC of this Pmb derivative is at 8 μg/ml (Fig. 3A). At sub-MIC concentrations of 0.4× and 0.8× MIC, growth resumed at later times, 180 and 450 min, respectively, indicating the emergence of tolerance similar to what we observed with nonfluorescent analog (Fig. 3A). Pmb_fl_ (0.5× MIC) rapidly localized to bacterial membranes (Fig. 3B), with the majority of WT cells (97.8 ± 0.33%) showing positive staining within 30 min (Fig. 3, B to D, and fig. S6A). Pmb_fl_ signal decreased significantly over time (80.6 ± 1.88% by 300 min; *P* = 0.001) (Fig. 3, B to D, and fig. S6, A and B), independent of photobleaching. We noted that before membrane saturation, Pmb_fl_ fluorescence appeared enriched at high-curvature sites, including poles and septa (Fig. 3B).

We monitored *rcsA-gfp* expression in WT cells exposed to Pmb_fl_ and observed activation of the RcsA-dependent membrane stress response at both the population (Fig. 3E) and single-cell levels (Fig. 3, F and G) across multiple exposure times. Using two-color imaging and flow cytometry, we quantified Pmb_fl_ binding and *rcsA-gfp* expression after 30, 90, and 300 min. After 30 min, *rcsA* induction colocalized with membrane-bound Pmb_fl_ in 11 ± 1% of cells, indicating that a subset of bacteria that bind Pmb_fl_ already exhibits activation of the envelope stress response (Fig. 3F and fig. S6A). At this early time point, most bacteria (87 ± 1.3%) bound Pmb_fl_ while maintaining low *rcsA* activity, suggesting that binding precedes detectable stress induction. Single-cell microscopy supported these findings but revealed higher apparent colocalization, consistent with the method’s increased sensitivity to weaker signals (Fig. 3G). At later time points, the fraction of dual-labeled *rcsA-gfp*^+^/Pmb_fl_^+^ cells increased (20 ± 0.4%) (Fig. 3F and fig. S6B), indicating that membrane-associated Pmb_fl_ correlates with activation of the envelope stress response. In parallel, a growing subpopulation of cells lacked both signals (16 ± 1.4%), consistent with adaptation and stress exit (Fig. 3, F and G, and fig. S6B). While Pmb_fl_ appears less efficient at triggering the stress response compared with native Pmb, it remains a relevant and effective tool for tracking antibiotic localization and linking Pmb binding with membrane damage during tolerance development.

### EV production quickly sequesters Pmb_fl_ and facilitates antibiotic clearance from the bacterial membranes

**Fig. 4.**
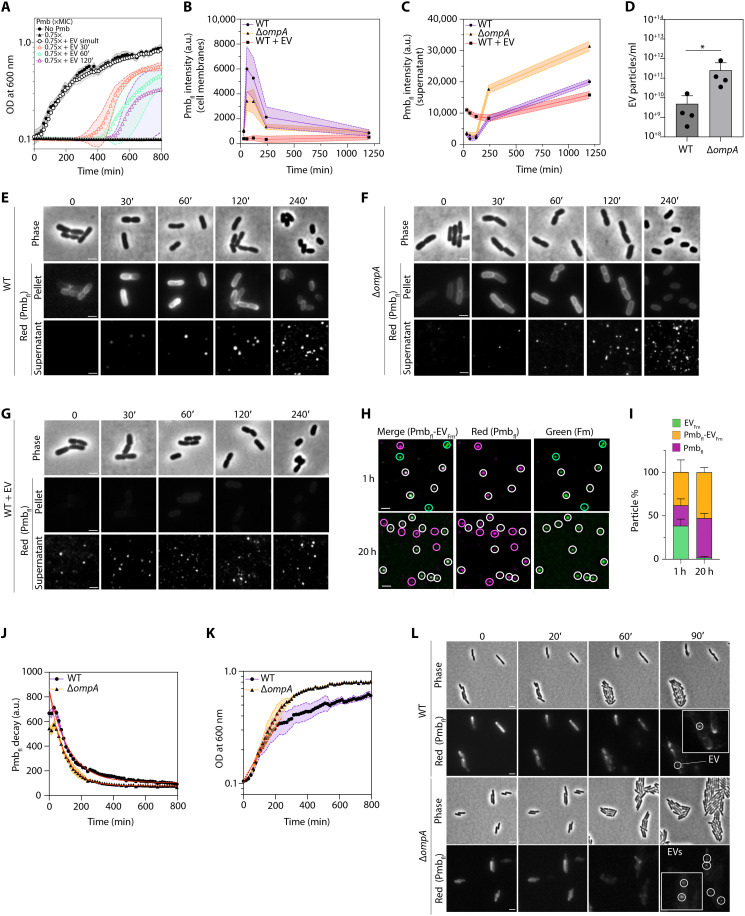
Vesiculation aids in trapping and clearing Pmb from bacterial membranes. (**A**) Growth curves of WT cells exposed to Pmb (0.75× MIC) with simultaneous or delayed (30 to 120 min) addition of purified EVs. Data show means ± SD (*n* = 6). (**B** and **C**) Time-course quantification of Pmb_fl_ in cell membranes (B) and filtered supernatants of WT, Δ*ompA*, and WT supplemented with purified EVs. Fluorescence was normalized to cell density [optical density (OD) at 600 nm]. Data show means ± SD (*n* = 3). Statistical analyses are described in Materials and Methods. a.u., arbitrary units. (**D**) EV production (in particles per milliliter) in WT and Δ*ompA*. Data show means ± SD (*n* = 4); Mann-Whitney test (*P = 0.02). (**E** to **G**) Fluorescence microscopy time course showing Pmb_fl_ insertion into cell membranes and release into free form or EV-associated particles in the supernatant (WT, Δ*ompA*, and WT + EVs). Phase-contrast and TRITC (purple, Pmb_fl_) images are shown. Membrane-associated Pmb_fl_ appears as fluorescent cell contours, while free or EV-bound Pmb_fl_ appears as bright puncta. Scale bars, 2 μm. (**H** and **I**) Supernatant imaging after 1 and 20 hours of growth with Pmb_fl_ (0.5× MIC). Free Pmb_fl_ is detected in the TRITC (purple) channel, EVs in the FITC (green, Fm1-43) channel, and Pmb_fl_-EV complexes in both channels. Scale bars, 1 μm. Quantification is shown in (I). (**J**) Decay of membrane-associated Pmb_fl_ following antibiotic removal. Time constants (τ) were 120.6 ± 2.9 min (WT) and 88.3 ± 1.6 min (Δ*ompA*). (**K**) Growth recovery after Pmb_fl_ removal following 30-min exposure. Growth rates determined from semilog regression fits to the exponential growth phase were 0.0022 and 0.0029 min^−1^ for WT and Δ*ompA*, respectively. (**L**) Single-cell time-lapse microscopy showing Pmb_fl_ decay and EV detachment following Pmb removal. Scale bars, 2 μm. Movies are provided in the Supplementary Materials (movies S1 and S2).

Adding pure EVs in a delayed manner (30, 60 or 120 min following Pmb addition) to the culture enabled growth restoration and emergence of drug tolerance, although with a longer lag phase before growth resumption (Fig. 4A). Because Pmb binds membranes within minutes, cells exposed for 60 min before EV addition have already sustained substantial membrane damage. The ability of late-added EVs to rescue growth suggests that EV-mediated tolerance mechanisms extend beyond simple sequestration or decoy effect.

To investigate these mechanisms, we tracked Pmb_fl_ turnover, encompassing membrane insertion and removal, in a time-course experiment. Cells were incubated with Pmb_fl_ and sampled at 0, 30, 60, 120, 240, and 1200 min. At each time point, cells were centrifuged, and Pmb_fl_ fluorescence was measured in the pellet fractions (cell membranes) (Fig. 4B) and in the supernatants (free-form and associated with EVs) (Fig. 4C). These experiments were performed using WT cells, an Δ*ompA* mutant that hypervesiculates because of loss of the structural outer membrane protein OmpA and produces ∼50× more EVs than WT (*P = 0.02; Fig. 4D), and WT cells supplemented with purified EVs at near-physiological levels. No growth and survival differences were observed in WT and Δ*ompA* strains in absence of treatment (fig. S7, A and C). Snapshot imaging of single cells further captured Pmb_fl_ redistribution dynamics across conditions (Fig. 4, E to G). In WT cells, Pmb_fl_ rapidly accumulated in the membranes, peaking at 30 min before gradually declining (Fig. 4, B and E). By 240 min and at later time points, Pmb_fl_ increasingly appeared in the supernatant (Fig. 4, C and E). Dual labeling with Fm1-43 showed that at 1 hour, 38 ± 14% of Pmb_fl_-positive particles were associated with EVs, while 38 ± 7.5% were Pmb-free EVs and 23 ± 7.5% were free Pmb_fl_ species (Fig. 4, H and I). At 20 hours, Pmb-free EVs decreased (2 ± 1.1%), whereas free form of Pmb_fl_ and EVs associated with Pmb_fl_ accumulated in the supernatant (45 ± 5.5% and 53 ± 5.4%, respectively) (Fig. 4, H and I). These results suggest that (i) EV populations are heterogeneous and dynamically change over time and (ii) there is a selective redistribution of Pmb_fl_ into EVs along with some passive dilution over time rather than uniform membrane loss.

We investigated Pmb_fl_ trapping and clearance via vesiculation and found that these processes were enhanced in hypervesiculating Δ*ompA* cells (Fig. 4, B, C, and F). In this strain, Pmb_fl_ accumulation in the supernatant increased from 120 min onward (*P* = 0.0004) (Fig. 4, C and F), correlating with a decrease in Pmb_fl_ signal in cell membranes (*P* = 0.04) (Fig. 4, B and F). In contrast, simultaneous addition of purified EVs and Pmb_fl_ led to rapid antibiotic neutralization by EVs within minutes (Fig. 4, C and G) and significantly reduced Pmb_fl_ access to the cell membranes at 60 min relative to WT (*P* = 0.01) with reduced membrane association maintained throughout the experiment (Fig. 4, B and G), confirming the EV-mediated decoy effect reported previously by other groups (*28*–*31*).

To examine the role of vesiculation in membrane repair and growth recovery, we monitored Pmb_fl_ decay and cell growth following Pmb_fl_ removal (Fig. 4, J to L). WT and Δ*ompA* cells were exposed to sub-MIC Pmb_fl_ for 60 min, then washed to remove extracellular fluorescent antibiotic and EVs, and resuspended in fresh medium. Fluorescence associated with cell pellets was used to quantify membrane-associated Pmb_fl_ over time. Pmb_fl_ clearance from membranes was slower in WT cells (τ = 120.6 ± 2.9 min) than in Δ*ompA* cells (τ = 88.3 ± 1.6 min). Although this difference had little effect on early growth recovery, it resulted in enhanced late-stage growth in Δ*ompA* cells (Fig. 4K), as supported by our microscopy snapshots (Fig. 4L). WT cells exhibited stronger mid-exponential growth inhibition (*t* = 90 min; Fig. 4, K and L, and movie S2).

Together, time-lapse imaging showed that Pmb_fl_ decay coincided with division restart, microcolony formation, and EV release (from ∼60 min; movies S1 and S2). In line with these growth dynamics, Δ*ompA* displayed a growth advantage under sub-MIC Pmb conditions (fig. S7, A to D), whereas hypovesiculating strains showed mild to pronounced lag phase (fig. S7E). None of the strains grew at 1× MIC (fig. S7, B and F).

### EV uptake supports membrane repair and enhances growth recovery of stressed bacteria

**Fig. 5.**
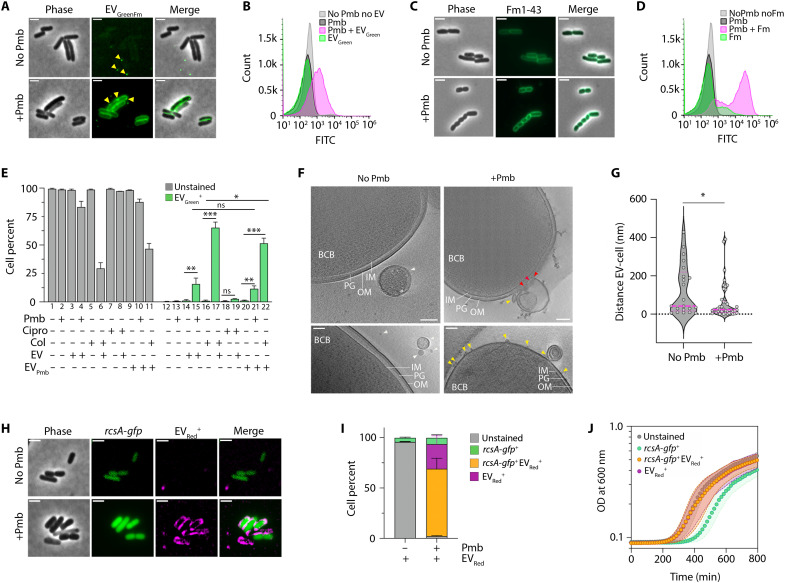
Uptake of fluorescently labeled EVs by *E. coli* membranes facilitates recovery of stressed bacteria. (**A**) Fluorescence microscopy images showing uptake of labeled EVs (EV_Green_) by WT cells exposed to Pmb (0.5× MIC, 30 min) or left untreated. Occasional free or detaching EVs are visible (yellow arrowheads). Phase-contrast and FITC images are shown. Scale bars, 2 μm. (**B**) Representative flow cytometry plots (50,000 events per sample) corresponding to (A). (**C**) Control images showing staining with Fm1-43 dye alone in the absence or presence of Pmb. (**D**) Representative flow cytometry plots corresponding to (C). (**E**) Quantification of EV_Green_ and EV_PmbGreen_ (Pmb-loaded EVs) uptake in the presence of membrane-active antibiotics (Pmb and colistin) or a nonmembrane-targeting antibiotic (ciprofloxacin), all at 0.5× MIC. Data show means ± SD (*n* = 3; 10,000 to 50,000 events per sample). Statistical significance (Welch’s *t* test; ns, *P* > 0.05; *P ≤ 0.05, **P ≤ 0.01; ***P ≤ 0.001). (**F**) Cryo–electron micrographs of EV interactions with *E. coli* cells cultured without Pmb or with Pmb. Bacterial cell bodies (BCBs), outer membrane (OM), inner membrane (IM), peptidoglycan (PG), and EVs in proximity (white arrowheads), adhering (yellow arrowheads), or fused (red arrowheads) to the outer membrane are indicated. Scale bars, 100 nm. (**G**) Quantification of EV-membrane distances measured using IMOD (no Pmb: median = 42 nm, *n* = 21; +Pmb: median = 23 nm, *n* = 42; *P = 0.02, Welch’s *t* test). (**H**) Two-color fluorescence imaging of Pmb-stressed *rcsA-gfp* cells taking up EV_Red_. Scale bars, 2 μm. (**I**) Flow cytometry quantification of EV_Red_ uptake (50,000 events per sample, *n* = 3). (**J**) Growth recovery of sorted cell subpopulations following Pmb removal. Data show means ± range (*n* = 2; six technical replicates).

In the following experiments, we examined bacterial uptake of EVs and its effect on growth recovery. To test this, we purified fluorescently labeled EVs using the lipophilic dye Fm1-43, which integrates into the lipid bilayer of the EV membrane. For these experiments, WT cells were pretreated with Pmb, incubated with fluorescent EVs (EV_Green_), and washed by centrifugation. Combining microscopy, flow cytometry, and cryo–electron tomography, we provide the first evidence that EVs can bind to and be incorporated into the cell membranes of *E. coli* upon Pmb exposure (Fig. 5, A and B, and fig. S8). In the absence of antibiotics, EV_Green_ uptake was minimal (1.2 ± 0.8%) in single cells (Fig. 5, A and B, and fig. S8). While membrane staining with Fm dye alone was uniform across the majority of Pmb-treated cells (Fig. 5, C and D), EV uptake occurred in discrete membrane patches and only in a subset of cells, revealing heterogeneity among EV recipients (Fig. 5, A, B, and E). EV_Green_ uptake increased significantly in cells challenged with membrane-targeting antibiotics, including Pmb (15 ± 5%) and colistin (65 ± 4.9%) (Fig. 5E, lanes 15 and 18, and fig. S8), but not with ciprofloxacin (2.5 ± 0.2%), a DNA replication inhibitor (Fig. 5E, lanes 19, and fig. S8). We also observed that EVs loaded with Pmb, slightly smaller in diameter (fig. S9, A and B), were also taken up by Pmb- and colistin-treated cells (10 ± 2.5% to 51 ± 4.5%, respectively) (Fig. 5E, lanes 21 and 22, and fig. S8, E to H), indicating that both Pmb-free and Pmb-loaded EVs have an affinity for damaged membranes. Uptake differences between Pmb-free and Pmb-loaded EVs were not significant for Pmb (lanes 15 to 21) but significant for colistin (lanes 17 to 22) (*P* = 0.014). Similar uptake was observed across dyes and with Pmb_fl_-loaded EVs (65% versus 0.6% uptake without Pmb) (fig. S9, C to H).

In line with these observations, cryo-EM revealed Pmb-dependent EV-cell interactions. In untreated cells, EVs remained spatially separated from the outer membrane, whereas Pmb exposure promoted close EV-membrane proximity and apparent fusion at discrete sites (Fig. 5F, fig. S10, and movies S3 and S4). Quantitative analysis confirmed reduced EV-membrane distances in Pmb-treated cells (Fig. 5G and fig. S10), supporting a model in which membrane damage facilitates EV docking and incorporation.

Next, we asked whether stressed bacteria (*rcsA-gfp*^+^) are more likely to take up EVs compared to nonstressed individuals (*rcsA-gfp*^−^). We used flow cytometry and our two-color imaging setup to monitor envelope stress (*rcsA-gfp*) and EV uptake (EV_red_) in single cells (Fig. 5, H and I, and fig. S9GH). Our data revealed that EV uptake was enriched in the stressed subpopulation (72 ± 5.8%), whereas nonstressed cells showed lower uptake (19.7 ± 0.8%) (Fig. 5I). The latter could represent dead cells (consistent with our microscopy images in Fig. 5H) or persister-like cells that failed to trigger a stress response yet retain membranes capable to take up EVs. We also identified a small subset of stressed cells (5.6 ± 4.2%) that did not efficiently take up EVs (Fig. 5I). These results suggest that the physiology of the recipient cells plays a critical role in determining EV uptake under stress conditions.

Last, to determine whether EV uptake contributes to the development of Pmb tolerance, we monitored EV-patched and unpatched subpopulations’ growth recovery. We cell-sorted fluorescently defined subpopulations, *rcsA-gfp*^+^ (green), EV_Red_^+^ (purple), and double-positive *rcsA-gfp*^+^EV_Red_^+^ (orange), as well as an unstained subpopulation representing cells expressing very low *rcsA-gfp* or not responsive to both Pmb and EV_Red_ (fig. S11). We isolated these subpopulations from gated *rcsA-gfp* cells under Pmb stress, exposed to purified EV_Red_^+^, and compared their growth recovery following Pmb removal (Fig. 5J). The sorted fractions consisted of EV-patched cells (18% total, with ∼12% stressed and ∼5% nonstressed) and nonpatched cells (73% total, with ∼40% stressed and ∼30% nonstressed) (fig. S11). Notably, EV-patched subpopulations, irrespective of their stress state, displayed early growth recovery, entering exponential phase at ∼250 min after an initial lag, similar to unstained cells. In contrast, non-EV–patched stressed cells exhibited a prolonged lag phase and resumed growth only after ∼400 min (Fig. 5J). These results indicate that EV uptake enhances the capacity of stressed cells to recover from Pmb exposure.

Together, these findings demonstrate that stress-induced EVs act as a membrane patch by adhering and/or fusing with damaged cell membranes. EV uptake by *E. coli* is selective, antibiotic dependent, and shaped by the physiological state of recipient cells and improves recovery after Pmb stress.

## DISCUSSION

The rising threat of antibiotic-resistant bacteria has heightened interest in AMPs such as polymyxins, which remain effective against many MDR Gram-negative bacteria (*1*, *9*). Polymyxins, such as Pmb and colistin, disrupt the bacterial membrane by targeting its LPS layer. However, bacteria have evolved strategies to tolerate or resist these effects (*7*). EVs play a key role in bacterial adaptation by transferring genetic material, proteins, and metabolites, aiding survival under stressors such as antibiotics (*27*). EVs can act as decoys to sequester polymyxins, reducing their efficacy (*28*–*33*). Despite these roles, the mechanisms by which EVs interact with bacterial membranes in response to antibiotic stress remain unclear, emphasizing the need for alternative approaches to studying the complex interplay between EVs, bacteria, and antibiotics.

**Fig. 6.**
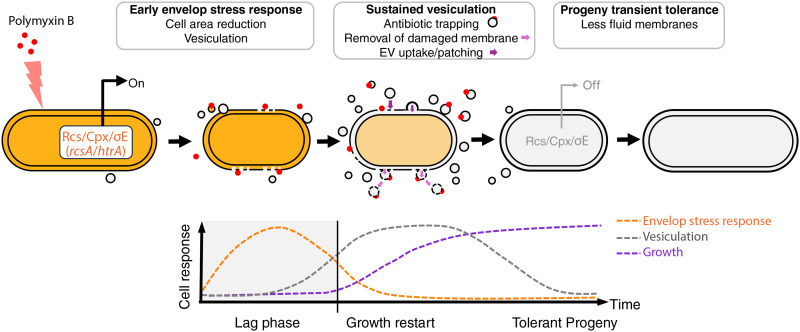
Stress-conditioned adaptation of bacteria to sub-MIC Pmb. Sub-MIC Pmb rapidly triggers Cpx/σE/Rcs envelope-stress responses during lag phase, leading to cell compaction, reduced exposed membrane surface, and early membrane remodeling. These responses stabilize the envelope and reduce permeability, while a burst of vesiculation is coupled to this early remodeling and contributes to the trapping and removal of damaged or antibiotic-loaded membrane material. As exposure continues, sustained vesiculation produces both Pmb-free and Pmb-loaded EVs and supports ongoing damage control by shedding and/or patching compromised membrane regions. Although membrane compaction dominates early membrane remodeling, EV release contributes more modestly initially but persists throughout adaptation, together promoting growth recovery and the emergence of transiently tolerant populations.

In this work, we combined single-cell and population-level approaches to track in real-time interactions between *E. coli*, EVs, and fluorescent Pmb during envelope stress, while monitoring stress responses, drug dynamics, vesicle exchange, and cell recovery. Our results support a biphasic adaptive response to sub-MIC Pmb (Fig. 6). In the early phase, Pmb rapidly triggers Cpx/σE- and Rcs-dependent stress responses, promoting cell compaction and initial membrane remodeling reducing exposed surface area. Concomitantly, cells release EVs that both act as decoys for Pmb and repair damage by exporting or patching Pmb-damaged membrane regions. As stress signaling diminishes, cells enter a second, Cpx/σE- and Rcs-independent phase in which vesiculation continues, mediating long-term membrane detoxification and repair, thereby providing population-level protection. Together, early stress-driven membrane changes and sustained EV-mediated detoxification and reorganization result in reduced permeability and increased rigidity in adapted progeny cells, supporting growth recovery and transient tolerance. These findings reveal previously unrecognized roles for EVs in tolerance to membrane-active antibiotics.

Exposure to Pmb triggered an immediate envelope-stress response involving both the Rcs phosphorelay (*rcsA*) and Cpx/σE (*htrA*), consistent with rapid outer membrane disruption. Pmb binding correlated with membrane damage load, as higher Pmb concentrations increased stress-reporter induction (Fig. 2I) and colocalization of Pmb_fl_ with the *rcsA-gfp* reporter over time (Fig. 3F). Within the first hour, cells exhibited significant surface area loss [Δ =7.62 μm^2^ (3D)] (Fig. 2D and table S3), reflecting global membrane remodeling. Cell compaction under envelope stress has not been systematically reported for membrane-active antibiotics; however, recent work has shown that *E. coli* bacteria sense and respond to physical changes in envelope dimensions, such as widening, via conserved pathways such as Rcs (*46*). Our results evidence a previously unidentified link between envelope stress and cell size reduction during antibiotic binding. This remodeling accounts for most early membrane compaction as we calculated that ∼1% of the ∼26% membrane lost during the first hour was redistributed in EVs (table S3). Thus, the bulk of membrane reduction reflects internal restructuring and cell shape reprogramming rather than large-scale shedding of membrane material. Despite representing a small fraction of total membrane loss at early stage, we showed that sustained vesiculation was functionally important to withstand sublethal Pmb concentrations. As exposure continues, vesiculation produces both Pmb-free and Pmb-loaded EVs (Fig. 4, H and I) and supports ongoing damage by removing (Fig. 4, B, C, and E) or patching (Fig. 5, A and B) compromised membrane regions and enabling the emergence of transiently tolerant populations (Figs. 1A and 5J).

Pmb_fl_ interacts with bacterial membranes, where it can be retained, released, or redistributed. Early dual-label imaging (Fig. 4, H and I) showed that particles released into the supernatant are associated with EVs, indicating selective shedding of drug-enriched or damaged membrane microdomains. As cultures enter late exponential growth, when vesiculation persists (Fig. 2A), Pmb_fl_-EVs accumulate, suggesting that actively dividing cells use EV production to accelerate drug removal. Pmb_fl_ was also detected in EV-free fractions (Fig. 4, H and I), indicating that detoxification involves both EV-mediated export and EV-independent redistribution. These results reveal dynamic Pmb behavior at the membrane: It can be retained in membranes or vesicles or released via vesiculation or diffusion. We also cannot exclude that released free Pmb may subsequently reassociate with EVs, supporting a decoy-driven detoxification even at later stages.

Our results showed that EVs can fuse with damaged bacterial membranes. EV patches appeared as discrete fluorescent foci along the cell envelope, pointing to direct EV-membrane interactions (Fig. 5A). Uptake was inherently heterogeneous: Depending on the antibiotic, only 15 to 60% of cells showed evidence of EV uptake, and the intensity of EV-patching signals varied, likely resulting from cell-to-cell differences in membrane damage and repair capacity. The EV population itself was also heterogeneous (Fig. 4, H and I), comprising both Pmb-free vesicles and Pmb-loaded vesicles—the latter displayed a smaller mean size (fig. S9B), likely more fusogenic because of higher curvature (*47*). Fluorescence microscopy and flow cytometry showed that both EV types could patch recipient membranes with similar efficiency under sub-MIC Pmb (Fig. 5E and fig. S9F). Although we were unable to capture many intermediate stages of vesicle fusion, cryo-EM supported our single-cell observations by capturing a number of EVs adherent to and merging with the outer membrane during Pmb stress (Fig. 5F, fig. S10, and movie S4). To our knowledge, this represents the first direct real-time visualization of bacterial EV fusion at the single-cell level using complementary approaches. Future correlative studies may reveal whether specific EV subpopulations, defined by size, curvature, or lipid composition, differ in fusogenic potential.

Functionally, cells receiving EV patches resumed growth faster than those without, underlying a role of EV-mediated membrane repair. In addition, cells with moderate to severe envelope damage are primed for higher EV uptake (Fig. 5, H and I). Variability in growth of the patched cells (Fig. 5J) likely arise from differences in EV composition and size, as well as the degree of recipient cell damage. In addition, the observation that unstained bacteria recovered growth as efficiently as stressed, patched cells is puzzling. These cells may represent a subpopulation experiencing minimal envelope stress or having intrinsic tolerance to Pmb, allowing growth recovery without detectable EV uptake. Determining whether this repair mechanism is random or selective and how donor and recipient traits shape EV exchange will deepen our understanding of EV-mediated communication and its role in antibiotic tolerance.

Last, our results highlight that bacterial tolerance is shaped by membrane structure and vesiculation. Δ*ompA* mutants, which produce ∼50-fold more EVs than WT (Fig. 4D and table S2), accumulated less Pmb_fl_, survived Pmb stress better, and recovered growth more rapidly than WT cells (Fig. 4, B, C, and J to L). A recent study has shown that OmpA interacts via its C-terminal periplasmic domain with periplasmic components, including peptidoglycan and the Rcs system regulator RcsF (*48*). Thus, loss of OmpA alters membrane organization by disrupting peptidoglycan interactions, rendering the outer membrane more dynamic and permeable, which may accelerate membrane repair and enhance Pmb clearance into EVs. In contrast, hypovesiculating mutants (Δ*wzzE* and Δ*nlpA*) survived low-stress conditions better but grew poorly under mild Pmb (fig. S9, E and F). Although Δ*ompA*, Δ*nlpA*, and Δ*wzzE* mutations change vesiculation by disrupting outer membrane-peptidoglycan tethering and altering enterobacterial common antigen (ECA)/LPS structure, respectively (*49*–*51*), and may have additional pleiotropic effects on membrane stress, the precise mechanisms by which they influence Pmb tolerance remain unclear.

The EV-mediated bacterial response to membrane injury shares similarities with eukaryotic strategies for coping with membrane damage (*52*). McNeil *et al.* (*53*) and Davenport *et al.* (*54*) demonstrated that large cells (i.e., echinoderm and frog oocytes) use membrane patches, induced by a local increase in Ca^2+^ and resulting from the fusion of small cytoplasmic vesicles with each other, to create a continuous membrane plug at the wound site along with the plasma membrane. More recently, Bussi *et al.* (*55*) evidenced a remarkable mechanism of membrane wound repair in human-induced pluripotent stem cell–derived macrophages, where stress granules act as membrane plugs to stabilize ruptured endolysosomal membranes and facilitate their repair. These studies highlight how vesicles and stress-related structures can contribute to cellular membrane repair, ensuring cell survival under adverse conditions.

In conclusion, this study reveals previously uncharacterized bacterial EV-mediated mechanisms in response to antibiotics through single-cell analyses. Future investigations are needed to clarify how these mechanisms are regulated over time, especially in response to varying environmental conditions and stressors. A more detailed understanding of the dynamic interplay between EVs and recipient cell physiology during antibiotic challenge could reveal new therapeutic targets to enhance antibiotic efficacy and limit the emergence of tolerance.

## MATERIALS AND METHODS

### Bacterial strains and growth media

The bacterial strains used in this study are listed in table S1. Precultures were grown in 2 ml of LB Lennox medium (pH 7.3) at 37°C with shaking at 150 rpm overnight. Experimental cultures were inoculated at a 1:100 ratio from precultures into LB Lennox and grown for 2.5 hours to reach the exponential phase [optical density (OD) at 600 nm = 0.4 to 0.5]. Antibiotic treatments included Pmb at concentrations ranging from 0.5 μg/ml (0.25× MIC) to 2 μg/ml (1× MIC), colistin at 0.75 μg/ml (0.25× MIC) to 3 μg/ml (1× MIC), tobramycin at 0.1 μg/ml (0.25× MIC) to 0.4 μg/ml (1× MIC), and ciprofloxacin at 10 ng/ml (0.2× MIC) to 50 ng/ml (1× MIC). Pmb_fl_ (Rhodamine-Pmb) was used at concentrations from 1.5 μg/ml (0.18× MIC) to 8 μg/ml (1× MIC). Unless otherwise noted, bulk experiments were conducted with Pmb and Pmb_fl_ at 0.5× MIC. All antibiotics were purchased at Sigma-Aldrich.

### EV production, isolation, and purification

A 1:100 dilution of an overnight culture of WT bacteria (or Δ*ompA* and Δ*rcsB* mutants when noted for increased EV production yields due to their hypervesiculation phenotype) was used to inoculate 50 ml of fresh LB medium. Cultures were grown for 1, 2, 4, 6.5, or 20 hours, with or without Pmb antibiotic, and cells were removed by centrifugation (5000 rpm for 30 min at 10°C; Eppendorf 5810 R centrifuge). The EV isolation protocol was adapted from a previous study (*37*). EVs were purified through filtration and ultracentrifugation. The supernatants were filtered through a 0.22-μm unit with a 50-ml syringe. To ensure the absence of bacterial contamination, 150 μl of the filtrate was plated onto LB agar and incubated at 37°C overnight; no colonies were observed after 24 to 48 hours. EVs were pelleted by ultracentrifugation at 41,000 rpm for 3 hours at 4°C (Optima L-80 XP ultracentrifuge, Beckman Coulter) using a 45Ti rotor. Supernatants were carefully and completely removed, and EV pellets were resuspended in 0.5 ml of freshly filtered phosphate-buffered saline [PBS; EDTA- and CaCl_2_-free (pH 7.5), 1×, filtered through 0.1-μm units], yielding a 100-fold concentration. Samples were stored at 4°C for no longer than 1 week (the maximum storage time with confirmed EV integrity and fluorescence) and validated for EV presence using fluorescence microscopy (Fig. 2B). We showed that the origin or nature of EVs had a negligible impact in our assays (fig. S5); therefore, for most experiments, we used EVs purified from *ompA* cells unless noted, as the yield of production was increased by about 50 times (Fig. 4D). For the assay with heated EVs, we purified EVs from the Δ*ompA* donor strain (20 hours of growth, concentration-normalized), and EVs were (i) untreated and stored at 4°C immediately after purification; (ii) heat treated and cooled at room temperature, incubated at 75°C for 30 min, and then allowed to cool to room temperature; or (iii) heat treated and stored cold, incubated at 75°C for 30 min, then placed immediately on ice, and stored at 4°C before use. Absolute EV concentrations and size distributions were determined using a nano–flow cytometer technology, widely applied for EV size distribution and purity assessment in the field of EVs (*56*–*59*) (fig. S9) at the Flow Cytometry facility, CR2T, Institut Pasteur. Means of concentrations of pure EV samples used in the study are reported in table S2; they ranged from 1.2 × 10^10^ EVs/ml (WT donor), 2.5 × 10^11^ EVs/ml (Δ*ompA* donor), and 1.4 × 10^11^ EVs/ml (WT + Pmb donor). For EV uptake assays, filtered supernatants of Δ*ompA* cell cultures were stained at 37°C for 20 min with lipophilic dyes [Fm1-43 (green) and Fm4-64 (red)] at a final concentration of 0.6 mg/ml. EVs were then pelleted by ultracentrifugation using the same protocol described above.

### Growth curves assays

We used a TECAN Infinite 200 PRO microplate reader for automated measurement of population growth curves with or without antibiotic stress, quantification of membrane stress reporter expression (*rcsA-gfp*), and quantification of Pmb_fl_ decay (insertion of Pmb_fl_ in cell membranes and EVs). Bacterial growth curve experiments were conducted at 37°C over 800 min unless noted with fluorescence reads at 474 nm (*rcsA-gfp*) or 560 nm (Pmb_fl_) when needed. Wells were inoculated with 2.5 μl of precultures into 150 μl of LB medium, supplemented as needed with antibiotics and/or EVs (∼1.2 × 10^9^; ∼160 EVs per cell) added concomitantly or with a delay (30, 60 or 120 min) to the wells.

Progeny growth curve analysis was performed in which cells previously exposed to subinhibitory Pmb (0.25× or 0.5× MIC) were passaged either in the presence of the same Pmb concentration or passaged without Pmb and subsequently reexposed to Pmb under identical conditions to assess adaptive growth responses. Growth was monitored by measuring OD at 600 nm over time to determine lag phase duration. Data were analyzed using GraphPad Prism 10.0.0 software (San Diego, CA, USA).

### Membrane property analysis

Naïve parental and Pmb-adapted progeny cells were exposed to 0.5× MIC for 1 hour before analysis. Membrane integrity, permeability, and fluidity were assessed using PI (1 μg/ml; Thermo Fisher Scientific, #P3566), Fm1-43 (1 μg/ml; Thermo Fisher Scientific, #T3163), and the polarity-sensitive probe NR12S (150 nM), respectively. PI reports on compromised membranes, Fm1-43 on membrane accessibility, and NR12S on lipid order, with relative LOR calculated as follows: (% green cells − % red cells) / (% green cells + % red cells); with % green cells as a proxy for $I_{530\text{nm}}$(ordered) and % red cells as a proxy for $I_{590\text{nm}}$(disordered). LOR (+1) indicates highly ordered, less fluid membranes, LOR (0) indicates mixed order, and LOR (−1) indicates more disordered, more fluid membranes. Pmb_fl_ (0.5× MIC) was used to examine antibiotic binding ability to cell membranes. Samples were analyzed by flow cytometry (as described further) and fluorescence microscopy (immobilized on pads, as described further).

### Whole-genome sequencing of adapted populations

To verify the presence of mutations in the Pmb-adapted populations, whole-genome sequencing of bacteria cultured in wells containing, LB only, LB + EVs, and LB + Pmb (1× MIC) + EVs (∼1.2 × 10^9^ added to the culture; ∼160 EVs per cell) was performed at the end of the growth curve run. Whole-genome sequencing was performed by the in-house Mutualized Platform for Microbiology (Paris, France) using the Nextera XT DNA Library Preparation Kit (Illumina Inc.), the NextSeq 500 sequencing system (Illumina Inc.), and the CLC Genomics Workbench 11 software (QIAGEN) for analysis. Coverage of at least 50× was obtained, guaranteeing a good quality sequence. No mutations (single-nucleotide polymorphisms or genetic rearrangements) were identified using the following tools breseq variant report v0.35 for the cells cultured with EVs, with and without Pmb. The reference sequence used to map the FASTQ files: *E. coli* strain K-12 MG1655 (*E. coli* reference genome) and RefSeq ID: GCF_000005845.1 – genome assembly: ASM584v2.

### Time course of Pmb_fl_ dynamics and decay assays

For the time course assay following Pmb_fl_ addition, we used 30 ml of cultures (dilution of 1:100 of precultures) grown in fresh LB to exponential phase (∼2.5 hours). Before Pmb_fl_ addition, a 1.5-ml sample (*t* = 0) was harvested, and the OD was measured. Then, the sample was centrifuged (10,000 rpm for 3 min), with the cell pellet immediately resuspended in PBS 1× (1.5 ml) and the spent medium containing vesicles collected by 0.22-μm filtration. Further samples were collected over time following the same protocol. For each sample, equal volumes of supernatant and cell pellet fractions were distributed in six replicates into a 96-well black flat-bottom plate, and OD at 600 nm and total fluorescence were measured at 560 nm using a TECAN Infinite M200 PRO microplate reader. All samples were imaged under the microscope on agar pads. For the time course assay following Pmb_fl_ removal (decay assay), we used 10 ml of cultures (dilution of 1:100 of precultures) grown in fresh LB to exponential phase (∼2.0 hours); then, Pmb_fl_ was added for 30 min. Before Pmb_fl_ removal, a 1.5-ml sample (*t* = 0) was harvested, and the OD was measured. The rest of the culture was centrifuged, and the pellet was resuspended in fresh LB. Red fluorescence signal and cell density were read at 560 and 600 nm, respectively, using a TECAN Infinite M200 PRO microplate reader. Samples were collected over time for microscopy imaging. In all analyses, the fluorescence signal was normalized to cell density. Data were analyzed using GraphPad Prism 10.0.0 software (San Diego, CA, USA). Statistical significance was measure across strains over time by Welch’s *t* test (pellets): WT versus *ompA* [*30 min, *P* = 0.013; 60 min, *P* = 0.03; not significant (ns) at 120 and 240 min], WT versus WT + EVs (*60 min, *P* = 0.01; ns at 30, 120, and 240 min), and *ompA* versus WT + EVs (**30 min, *P* = 0.009; *60 min, *P* = 0.02; *120 min, *P* = 0.04; *240 min, *P* = 0.02); Welch’s *t* test (supernatant): WT versus WT + EVs (ns at 30 and 60 min; **120 min, *P* = 0.0016; ns at 240 min), WT versus *ompA* (ns at 30, 60, and 120 min; **240 min, *P* = 0.001), and *ompA* versus WT + EVs (ns at 30 and 60 min; **120 min, *P* = 0.0017; 240 min, *P* = 0.001). A nonlinear one-phase exponential dissociation model was fitted to fluorescence decay data using points from 60 to 800 min to extract the time constant tau (τ) that characterizes how quickly Pmb_fl_ decays from membranes over time. Note that Pmb_fl_ fluorescence can differ depending on its environment: When bound to compact, protein-rich cell membranes, the signal is strongly quenched, whereas free, fragment-associated, or EV-bound Pmb_fl_ fluoresces much more brightly (information obtained from manufacturer). It should be noted that the supernatant signal includes Pmb_fl_ released both as free (nonvesicular) species and from EVs. Any apparent discrepancy in fluorescence intensity between the supernatant and cell membranes may reflect the behavior of the fluorophore and the presence of mixed Pmb_fl_ species.

### Microscopy imaging setup for bacterial cells, Pmb_fl_, and EV_fluo_

For all experiments, cell cultures were grown with or without sub-MIC antibiotics in liquid LB medium to mid-exponential phase and then transferred to 1.3% agarose-padded slides containing LB. A coverslip was placed on the agarose pad and sealed with a 1:1:1 mix of vaseline, lanolin, and paraffin to prevent evaporation. Imaging was performed immediately at 37°C using a Zeiss ApoTome inverted wide-field microscope for time-lapse analysis.

To study the interactions between Pmb_fl_, EVs, and bacteria, snapshot images were captured at intervals of 0, 30, 60, 120 and 240 min after adding Pmb_fl_ (0.5× MIC) to cultures. Images were taken with a Plan Apo 63× objective (numerical aperture = 1.4, +optovar 1.6×) and recorded using a Hamamatsu ORCA-Flash 4.0 v3 scientific complementary metal-oxide semiconductor camera (Institut Pasteur Imaging Facility, CR2T).

Pmb_fl_-stained fractions (cell pellet and supernatant) were imaged using two channels: red (560 nm) and phase contrast. Fluorescent EVs were imaged in the red channel [excitation/emission (Ex/Em) = 550/590 nm] when stained with the lipophilic dye Fm4-64 (Thermo Fisher Scientific, T3166). EVs labeled with Fm1-43 (Thermo Fisher Scientific, T3163) and stressed bacteria (*rcsA-gfp*) were imaged in the green channel (Ex/Em = 488/590 nm and Ex/Em = 488/520 nm, respectively). Pmb_fl_-bound EVs were imaged from filtered supernatants of WT bacteria exposed to Pmb 0.5× MIC for 1 and 20 hours. A volume of 0.8 μl of purified Pmb_fl_-bound EVs was immobilized on an agarose pad containing Fm1-43 dye and imaged. Only large vesicles are visible (>150 nm). Control pads with Pmb_fl_ or Fm1-43 alone were imaged in parallel. EV dual labeling images (EVOS M7000, Thermo Fisher Scientific, Olympus 100× objective), mean cell length, roundness, and area were measured from phase-contrast microscopy images (Zeiss Axio Observer 63× + Optovar 1.6×). Images were analyzed in FIJI software (*60*). Cell roundness was measured in FIJI using the fitted ellipse major axis, yielding a unitless metric (0 to 1) reflecting cell elongation versus circularity; values closer to 1 indicate more circular cells, and values closer to 0 indicate elongated shapes.

### Sample preparation for EV uptake analysis

WT, WT pPr*rcsA-gfp*, and Δ*ompA* cells were grown in 20 ml of LB Lennox medium at 37°C for 2.5 hours from 1:100 diluted precultures. A 0.5-ml aliquot of culture (10^8^ cells) was transferred into 2-ml Eppendorf tubes. When required, antibiotics [Pmb (0.5× MIC final), Pmb_fl_ (0.5× MIC final), ciprofloxacin (0.4× MIC final), or colistin (0.5× MIC final)] and EVs (10 μl of pure fraction at 10^10^ particles/ml, stained or unstained) were added to the 0.5-ml culture tubes. Tubes were incubated at 37°C with shaking for 30 min (tubes lay down with tape). For EV uptake experiments, fluorescently labeled EVs [∼1 × 10^10^ EVs (native) or EVs (Pmb-loaded); mean ratio of 72 EVs per cell; table S2] were added for 10 min immediately after antibiotic exposure (30 min), and the culture mix was put at 37°C with shaking. Then, the samples were centrifuged at 13,000 rpm for 4 min. Supernatants were carefully discarded to remove the drug and the EVs, and the cell pellets were resuspended in 0.5 ml of filtered 1× PBS (100-nm filter; Fisher scientific). Tubes were wrapped with foil before further analysis. Samples were imaged using a Zeiss Apotome inverted wide-field microscope [UtechS Photonic BioImaging (Imagopole)] for controls.

### Flow cytometry and cell sorting workflow

For flow cytometry, samples were diluted 1:10 and analyzed with a CytoFLEX flow cytometer S (Beckman Coulter, France), operated with the CytExpert 2.4.0.28 software (Beckman Coulter, France). The machine is equipped with 488-nm (50 mW) and 561-nm (30 mW) lasers. The 488-nm laser light was used for the detection of forward scatter (FSC) (488/8-nm band-pass), side scatter (SSC) (488/8-nm band-pass), with a double threshold on both parameters. The fluorescein isothiocyanate (FITC) fluorescence (513/26-nm band-pass) was measured using the 488-nm laser for excitation, and phycoerythrin (PE) (585/42/80-nm bandpass) was measured using the 561-nm laser. The fluidic system ran at a constant speed of 30 μl/min. Fluorescence intensity and cell counts (*N* = 10,000 to 50,000 for each sample) were measured using an automated method for diluted live bacterial cells. Cell Sorting was performed with the MoFlo Astrios (SUMMIT software version 6.3.1.16945) (Beckman Coulter, France) at 25 psi (172.4 kPa) with a 100 nM nozzle at ∼6000 events/s. The FSC and SSC were read logarithmically with the 488-nm laser. FITC fluorescence was read with the 488-nm laser (513/26-nm band-pass) and PE (579/16-nm band-pass) with 561-nm laser. Samples treated with Pmb and supplemented with fluorescent EVs were sorted and analyzed. Sorted populations (various cell counts, 3000 to 360,000 cells) included unstained cells, red (PE, EV-patched), green (FITC, *rcsA-gfp*^+^–stressed), and red + green (EV-patched *rcsA-gfp*^+^–stressed) cells. All data were processed using FlowJo software (FlowJo v10.10.0, Becton, Dickinson and Company, Ashland, OR, USA). For growth recovery experiment, cell counts were normalized across sorted populations when seeding the wells of the 96-well plate. Data were analyzed using GraphPad Prism 10.0.0 software (San Diego, CA, USA). For all experiments, representative histograms (FSC, FITC, or PE intensity) are available in the Supplementary Materials.

### Cryo–electron tomography

For sample preparation, an overnight culture (1 ml) was centrifuged, and the pellet was resuspended in 0.6 ml of fresh LB, with or without Pmb (0.5× MIC) for 60 min at 37°C. A 0.1-ml volume of pure EVs was added for 30 min. The tubes were centrifuged to remove excess EVs and drug, and the pellets were then resuspended in 1× PBS. A 4-μl volume of the sample mix was drop casted on glow-discharged Quantifoil R 2/2 on 200 gold mesh grids (Oxford/Quantifoil) and left to adsorb for 1 min. Cell density on the grid was then verified using an upright Zeiss Apotome microscope (bright-field channel) before the back blotting step against Whatman paper for 7 s. Cryo-fixation performed by plunge freezing at −180°C at 75% humidity in liquid ethane using a Leica EMGP (Leica, Austria). The grids were then immediately transferred for storage in liquid nitrogen before data collection.

Regarding the imaging protocol and equipment, dose-symmetric tilt series were collected on a 300-kV Titan Krios (Thermo Fisher Scientific) transmission electron microscope equipped with Falcon 4i direct electron detector (Thermo Fisher Scientific) and Selectris X imaging filter (Thermo Fisher Scientific). Tomography software (Thermo Fisher Scientific) was used to acquire tilt series with a tilt span of ±45° and an angular increment of 3°. The total electron dose was ∼120 electrons/Å^2^ and the pixel size at 3.101 Å. Tilt series were saved as separate stacks of .eer frames, subsequently motion corrected, and coarse aligned using IMOD software. In parallel with data collection, an on-the-fly reconstruction software Tomo Life (Thermo Fisher Scientific) was used to both judge the quality of acquired data and reconstruction. Further, aligned stacks were denoised using IsoNet for better visualization. We measured the distances between EVs and bacterial cell membranes on the acquired screenshots using IMOD image analysis software (*61*) following the software’s standard measurement tools.
