# Supplementary material for: Bacterial extracellular vesicles promote membrane repair and tolerance to polymyxin B
Source: Sci Adv. 2026 Jun 10;12(24):eadx6378. doi: 10.1126/sciadv.adx6378 (PMC13251856; doi:10.1126/sciadv.adx6378)
Supplement: Supplementary file 1 — Tables S1 to S3 Figs. S1 to S11 Legends for movies S1 to S4 [file sciadv.adx6378_sm.pdf]

Science Advances
AAAS

Supplementary Materials for

Bacterial extracellular vesicles promote membrane repair and tolerance to polymyxin B

Julia Bos *et al.*

Corresponding author: Julia Bos, julia.bos@pasteur.fr

Sci. Adv. **12**, eadx6378 (2026)
DOI: 10.1126/sciadv.adx6378

The PDF file includes:

Tables S1 to S3
Figs. S1 to S11
Legends for movies S1 to S4

Other Supplementary Material for this manuscript includes the following:

Movies S1 to S4

Table S1 – List of strains used in this study.

Strain name	Species/Genotype	Reference
wt	*E.coli* K12 MG1655 Wild-type (RefSeq accession no. NC_000913.3)	Lab collection
∆*ompA*	*E.coli* K12 MG1655 ∆*ompA::kan*	Lab collection
Horizon Discovery pPr *rcsA-gfp*	*E.coli* K12 BW25113 pP*rcsA-gfp (Kan)* Horizon Discovery *E.coli gfp* promoter collection # PEC3876-98156054	This study
Horizon Discovery pPr *htrA-gfp*	*E.coli* K12 BW25113 pPr *rcsA-gfp (Kan)* Horizon Discovery *E.coli gfp* promoter collection # PEC3876-98156054	This study
Horizon Discovery pPr *osmB-gfp*	*E.coli* K12 BW25113 pPr *rcsA-gfp (Kan)* Horizon Discovery *E.coli gfp* promoter collection # PEC3876-98156054	This study
wt pPr *rcsA-gfp*	*E.coli* K12 MG1655 (wt) pPr *rcsA-gfp (Kan)*	This study
wt pPr *htrA-gfp*	*E.coli* K12 MG1655 (wt) pPr *htrA-gfp (Kan)*	This study
wt pPr *osmB-gfp*	*E.coli* K12 MG1655 (wt) pPr *osmB-gfp (Kan)*	This study
ompA pPclfim	*E.coli* K12 W3110 (wt) ∆*ompA::kan pPclfim (spec)*	Ref (*38*)
UPEC	*E.coli* UPEC (wt) UGB554 CFP073	Gift from JM Ghigo's lab
BW25113	*E.coli* K12 BW25113 wild-type [∆(*araD-araB*)*567* ∆(*rhaD-rhaB*)*568* *∆lacZ4787* (::rrnB-3) *hsdR514 rph-1*]	Gift from JM Ghigo's lab
∆*nlpA*	*E.coli* K12 BW25113 ∆*nlpA::kan (KEIO collection #* OEC4987-213607683*)*	Gift from JM Ghigo's lab
∆*wzzE*	*E.coli* K12 BW25113 ∆*wzzE::kan (KEIO collection #* OEC4987-213607039*)*	Gift from JM Ghigo's lab

Table S2 – Concentrations of EVs and ratios EV counts per cell.

A

EV donor cell and growth condition			CFUs/ml	EVs/ml	Mean Ratio EV/cell (Physiological)
Exponential	wt	mean	2.8 E+08.	9.47 E+08	0.21
		stdev.	5.6 E+07	4.5 E+08	
	wt+Pmb	mean	3.2 E+07	1.4 E+10	436
	(0.4x)	stdev.	9.3 E+06	3.3 E+09	
	ompA	mean	4,69 E+08	n.d.	n.d.
		stdev.	1.9 E+07		
Stationary	wt	mean	3.17 E+09	1.24E+10	3.92
		stdev.	1.3 E+09	1.79 E+10	
	wt+Pmb	mean	2.1 E+09	1.4 E+11	67
	(0.4x)	stdev.	1.3 E+09	1.9 E+11	
	ompA	mean	2.3 E+09	2.55 E+11	111
		stdev.	8.0 E+08	1.17 E+12	

B

Addition of pure EVs	Mean Ratio EV/cell (cell growth assays)	Mean Ratio EV/cell (uptake assays)
EV_{ompA}	161	72
EV_{wt}	7.8	3.5
EV_{wt+Pmb}	91	41

Table S2 – Concentrations of EVs and ratios of EV counts per cell. A. Mean and standard deviations of cell counts (CFUs per ml) and EV concentrations (per ml) are reported across strains and conditions of growth (exponential phase, stationary phase, that are used in this study. EV concentrations were measured using a nano-flow cytometer (Nanofcm technology). Means of EV/cell ratio are indicated. **B.** Means of EV/cell ratio calculated for three types of pure EVs used in cell growth assays and EV uptake assays. The origin of the EVs is indicated (*ompA*, wt, wt+Pmb cells cultured overnight). Typically, for a growth assay in microplate, we used 5 µl EV_{ompA} or EV_{wt} or EV_{wt+Pmb} (normalized for concentration; from stationary phase culture) mixed with 2.5 µl wt cells (from stationary phase culture), and for the uptake assays, we used 40 µl EV_{ompA} or EV_{wt} or EV_{wt+Pmb} (normalized for concentration; from stationary phase culture) mixed with 0.5 ml of wt cells (exponential phase).

Table S3 – Quantitative analysis of membrane redistribution

Parameter / Condition	Measured value	Method
EV count (t1h + Pmb)	8.37×10^8 particles·mL⁻¹	NanoFcm (supernatant, corrected for 100x concentration)
Baseline EV count (t1h, no Pmb)	~5×10^6 particles·mL⁻¹	NanoFcm
EV mean diameter	63.5 nm (r = 0.0318 µm)	NanoFcm
EV mean surface area	0.01268 μm^2 per EV	Calculated ($4\pi r^2$)
Total EV membrane area	1.06×10^7 μm^2/mL	EV count x EV surface area
Mean cell length (L)	t0, L= 5.3 µm; t1h, L= 4.5 µm; t1h + Pmb, L= 3.2 µm	Phase-contrast microscopy (Zeiss Axio Observer 63x + Optovar 1.6x, FIJI segmentation)
Mean cell roundness (R)	t0, R= 0.25; t1h, R= 0.21 µm; t1h + Pmb, R= 0.37 µm	Phase-contrast microscopy (Zeiss Axio Observer 63x + Optovar 1.6x, FIJI segmentation)
Mean cell area (2D) (A)	t0, A = 4.897 μm^2; t1h, A = 3.977 μm^2; t1h + Pmb, A = 2.911 μm^2	Phase-contrast microscopy (Zeiss Axio Observer 63x + Optovar 1.6x, FIJI segmentation)
Mean cell area (3D corrected) (A)	t0, A = 34.9 μm^2; t1h, A = 28.4 μm^2; t1h + Pmb A= 20.8 μm^2	Cylindrical-rod model (3D/2D factor ≈ 7.14)
Δ cell area (2D)	t0=> t1h: 0.92 μm^2; t1h => t1h +Pmb: 1.067 μm^2; t0=> t1h +Pmb: 1.986 μm^2	FIJI image analysis
Δ cell area (3D corrected)	t0=> t1h: 6.57 μm^2; t1h => t1h +Pmb: 7.62 μm^2; t0=> t1h +Pmb: 14.18 μm^2	3D correction factor applied
Cell count (CFU/mL)	t0 = 7.01×10^7; t1h = 5.54×10^8; t1h + Pmb = 1.2×10^8	CFU plating
PI^+ cells (% lysis/death)	1 h no Pmb: 2%; 1 h + Pmb: 7.5%	Flow cytometry (PI staining)
Total cell membrane loss (t1=> t1+Pmb)	1.04×10^9 μm^2/mL	Δ3D per cell x cell count
EV membrane fraction (%) (t1=> t1+Pmb)	≈1.0% of total membrane loss	(EV membrane area ÷ total loss) x 100
Per-cell membrane loss (%)	t1h=>t1h+Pmb: 26.8%; t0=>t1h (no Pmb): 18.8%; t0=>t1+Pmb: 40.4%	Calculated from 3D-corrected areas

Table S3- Quantitative analysis of membrane redistribution. To assess membrane redistribution, we quantified total cell membrane per mL and the fraction incorporated into EVs after 1 h of Pmb treatment (0.5xMIC), time at which stress response, cell compaction and vesiculation are induced. We did the following measurements: total cell counts (CFUs/mL), 2D mean cell area (microscopy imaging) and 3D-corrected mean cell area (for a spherocylinder model), EV particle counts per mL of culture and EV mean size (Nanofcm). EVs were modeled as spheres and rod-shaped bacteria were modeled as cylinders with hemispherical ends (correction factor ≈ 7) to convert 2D projected areas into 3D membrane surface. From our numbers, only ~1% of the bacterial membrane lost during the first hour of Pmb exposure appears to be shed in EVs. Thus, EV production after 1 h of Pmb treatment accounts for only a small fraction of total membrane loss. The majority of membrane depletion likely occurs via internal remodeling and cell-shape reprogramming following induction of the membrane stress response, rather than wholesale redistribution into EVs. EV count and size were obtained by Nanofcm from supernatants of cultures exposed to Pmb, (0.5x MIC, 1 h). Cell counts were determined as CFUs/mL at t0, t1h ± Pmb. Mean cell length roundness, and area were measured from phase-contrast microscopy images (Zeiss Axio Observer 63x + Optovar 1.6x) and segmented in FIJI. The 2D projected areas were converted to 3D surface estimates using a cylindrical-rod model (cylinder + hemispherical ends; 3D/2D correction factor ≈7.14 for typical *E. coli* dimensions: width ~1 µm, length ~2.5 µm). Changing the *E. coli* dimensions to width ~0.7 µm, length ~2 µm changes the 3D/2D correction factor to ≈3.14 and only increase to ~1.79% of the bacterial membrane lost during the first hour of Pmb exposure. Cell death and lysis were quantified by propidium iodide staining (flow cytometry). CFU survival assays were also performed at t0, t1h ± Pmb.

From the numbers below, we estimated per-cell membrane loss (3D corrected): t 1=> t1+Pmb,

- EV count (t1h + Pmb) = 8.37 x 10^8 particles/mL
- EV mean surface area = 0.01268 μm^2 per EV
- Mean cell area (3D corrected) t0 = 34.9 μm^2; t1 = 28.4 μm^2; t1 + Pmb = 20.8 μm^2
- Δ cell area per cell (3D corrected) t1=>t1+Pmb = 7.62 μm^2 per cell
- Cell count (cells/mL)= 1.2 x 10^8 (t1+ Pmb); 5,54 x 10^8 (t1); 7.01 x 10^7 (t0)
- per-cell membrane loss (3D corrected): t1=>t1+Pmb: (28.4−20.8)/28.4=0.268(28.4 - 20.8)/28.4 = 0.268(28.4−20.8)/28.4=0.268, so **26.8%** loss.

From these other numbers below, we estimated the percent of the membrane lost during the first hour of Pmb exposure that appears in EVs:

- EV total membrane area = 1.06 x 10^7 μm^2/mL
- Number of survivors at t1+Pmb = 1.20x10^8 cell/mL
- Total cell membrane loss = 7.62 μm^2 × 1.20×10^8 = 9.14 x 10^8 μm^2/mL
- EV fraction = 1.06x10^7 / 9.14x10^8 = **1.16%**

Supplemental figures and captions

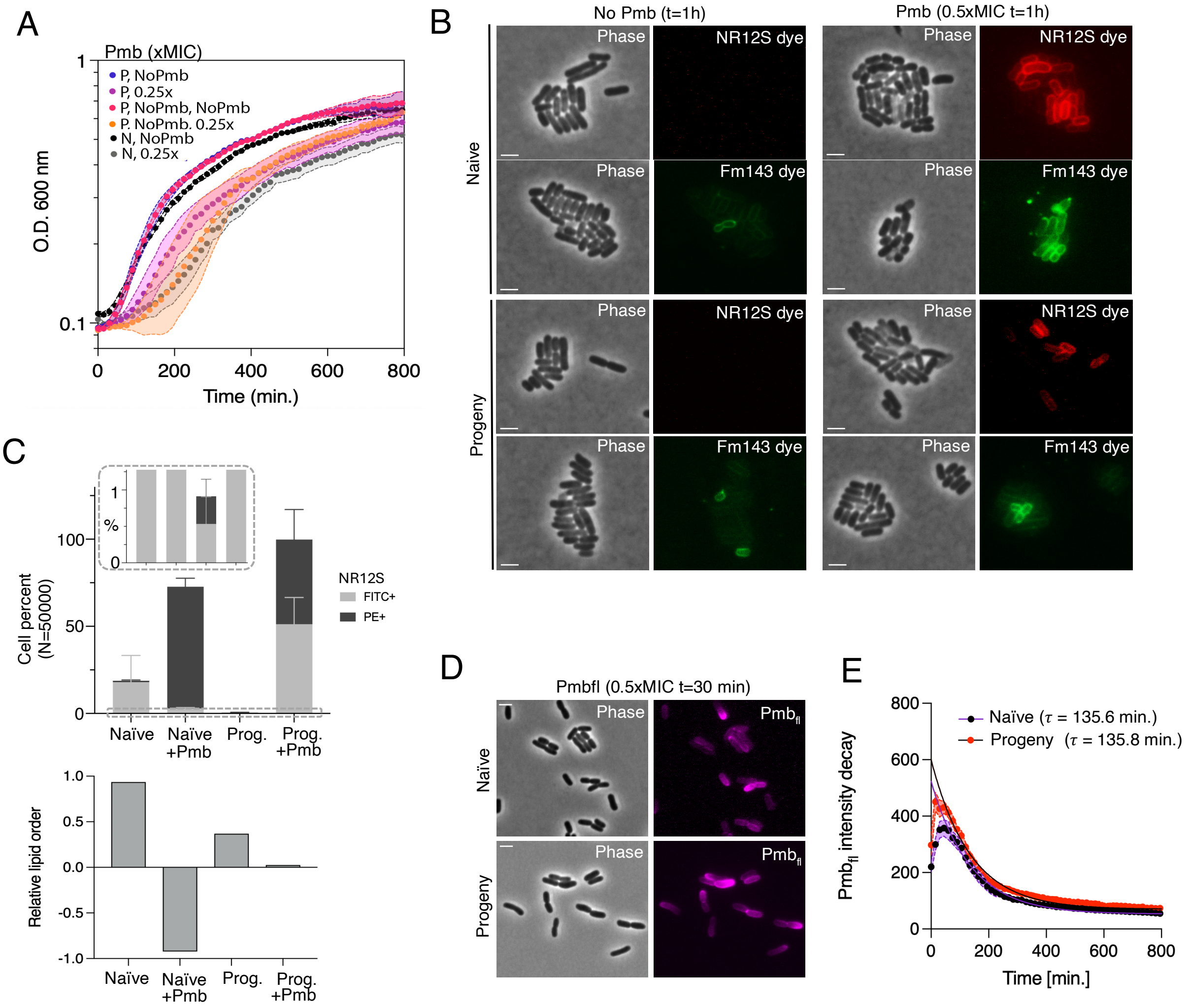

Fig. S1. Adaptive responses to Pmb and associated membrane property changes. (A) Growth curves of naïve and adapted cells cultured in the presence or absence of Pmb (0.25x MIC). Naïve cells (black/gray circles) had no prior Pmb exposure, whereas adapted cells (colored circles) were previously grown at 0.25x MIC Pmb. Cells were passaged with or without Pmb and, where indicated, re-exposed under identical conditions. Lag phase duration was used as a proxy for adaptive growth responses in progeny cells (n = 3). **(B)** Representative fluorescence microscopy images showing membrane properties of naïve and adapted progeny cells after 1 h exposure to Pmb (0.5x MIC). Fm1-43 was used to assess membrane permeability, and NR12S to report membrane fluidity (see Methods). Scale bar, 2 microns. **(C)** Flow cytometry quantification of NR12S emission in naïve and adapted progeny cells (derived from 0.5x MIC Pmb). Green and red-shifted emission populations were used to calculate lipid order ratio (LOR), defined as the ratio of blue (FITC, 520 nm; ordered lipids) to red (PE, 590 nm; disordered lipids) emission. Under Pmb stress, naïve cells showed a pronounced red shift (LOR=-0.92), indicative of increased membrane disorder, whereas adapted progeny exhibited reduced red-shifted emission and higher lipid order (LOR = 0.36). Data represent mean ± SD (n = 3). **(D)**

Representative microscopy images of Pmb_{fl} membrane insertion after 30 min treatment in naïve and adapted progeny cells (derived from 0.5x MIC Pmb). Scale bar, 2 microns. **(E)** Decay kinetics of membrane-associated Pmb_{fl} in naïve (black dots) and adapted progeny (red dots) cells. Data represent mean (n=2) and error range. Exponential fits yielded similar time constants (τ = 136.8 min and 135.6 min, respectively).

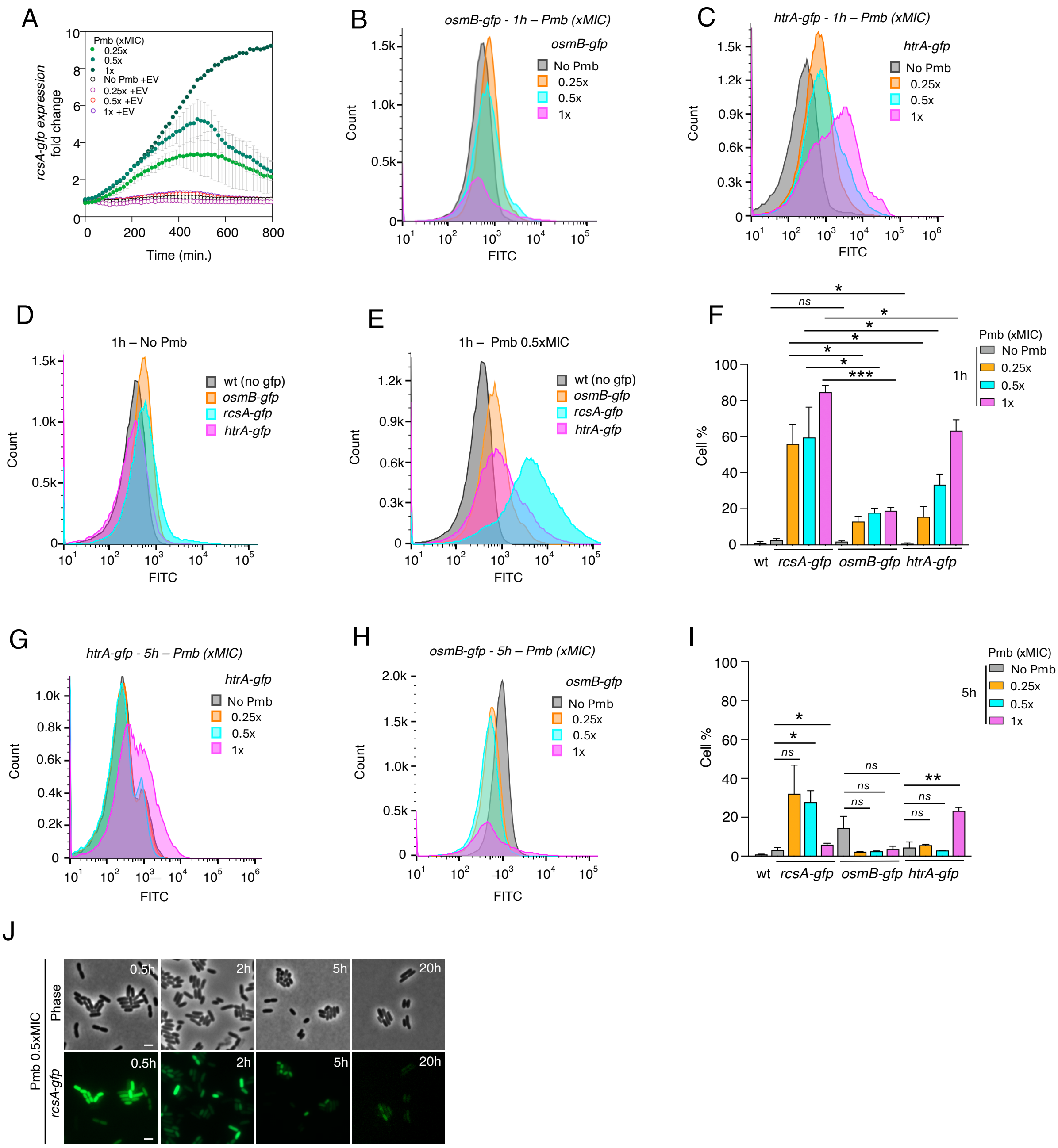

Fig. S2. Early activation of Rcs and σE envelope stress responses. (A) Population-level fold change in *rcsA-gfp* expression. GFP fluorescence was normalized to cell density (OD600) and to untreated controls. Simultaneous addition of Pmb (at the indicated concentrations) and EVs suppressed *rcsA-gfp* induction. Data represent mean ± SD (n = 3). **(B-F)** Single-cell flow cytometry analysis of envelope stress reporter expression for *osmB-gfp* (B) and *htrA-gfp* (C) after 1 h exposure to Pmb at 0.5x MIC, compared with *rcsA-gfp* expression in the absence of Pmb (D) and after 1 h of Pmb exposure at 0.25x, 0.5x, or 1x MIC. (E). Quantification is shown in (F).**(G-I)** Single-cell flow cytometry analysis of *htrA-gfp* (G) and *osmB-gfp* (H) expression after 5 h of Pmb exposure at 0.25x, 0.5x, or 1x MIC, with quantification including *rcsA-gfp* shown in (I). Across conditions, *osmB-gfp* showed weaker induction compared to that of *htrA-gfp*. In all plots (except *osmB-gfp* 5h, 1X, N<10000), N=50,000 events were collected per sample. Data represent mean ± SD (n = 3). Statistical significance (Welch's t-test) is indicated. **(J)** Representative phase-contrast and fluorescence microscopy images showing *rcsA-gfp* expression in wt single cells following exposure to Pmb (0.5x MIC) over time. Scale bar, 2 microns.

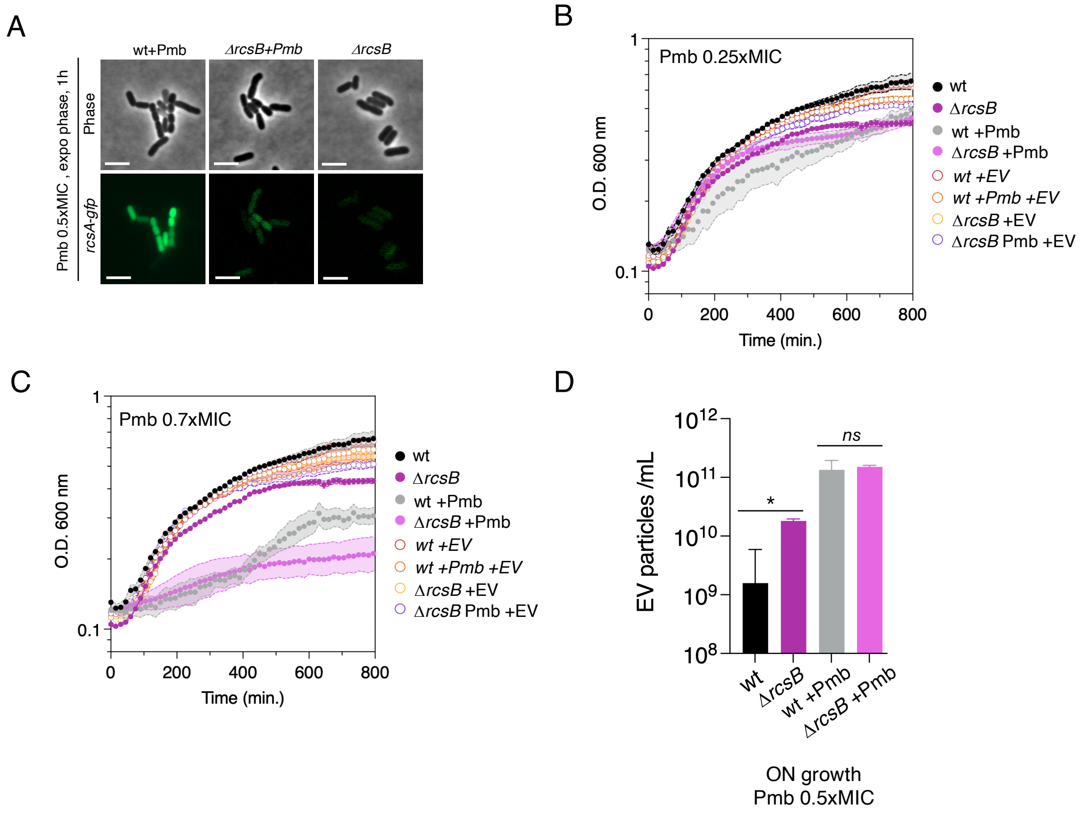

Fig. S3. Δ*rcsB* cells fail to induce *rcsA* and exhibit mixed sensitivity to Pmb. **(A).** Representative fluorescence microscopy images of *rcsA-gfp* expression in wt and Δ*rcsB* cells following 1 h exposure to Pmb (0.5x MIC). Scale bar is 4mm. **(B-C)** Growth curves of wt and Δ*rcsB* cells cultured in the absence or presence of Pmb at 0.25x MIC (B) or 0.7x MIC (C). Optical density at 600 nm (OD600) was measured over time. Simultaneous addition of Pmb (at the indicated concentrations) and EVs suppressed Pmb-induced growth defects in both strain backgrounds. Data represent mean ± SD (n = 3). **(D)** EV production (particles/mL) by wt and Δ*rcsB* strains grown overnight in the presence of Pmb (0.5x MIC), quantified using a nanoflow cytometer (NanoFcm). Data represent mean ± SD (n = 3). Statistical significance is indicated (Welch's unpaired t-test).

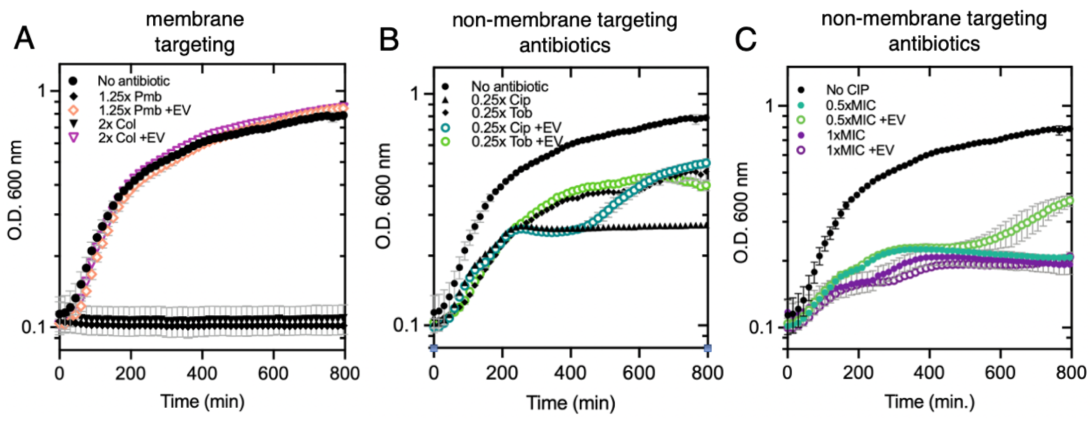

Fig. S4. Effect of pure EVs on wt strain growth with membrane-active and non-membrane active antibiotics. (A) Cells cultured with membrane-active antibiotics at high doses (1.25x MIC Pmb and 2x colistin) with or without EVs (from Δ*ompA* donor cells, concentration normalized to 2.5 $x10^{+9}$ EV/ml). **(B-C)** Cells cultured with non-membrane-active antibiotics at subMIC doses (0.25x (B) 0.5 xMIC ciprofloxacin (C) and 0.25x MIC tobramycin (B)) or MIC doses (1x MIC ciprofloxacin) (C), with or without EVs. In all plots data are shown as mean ± SD (n=3).

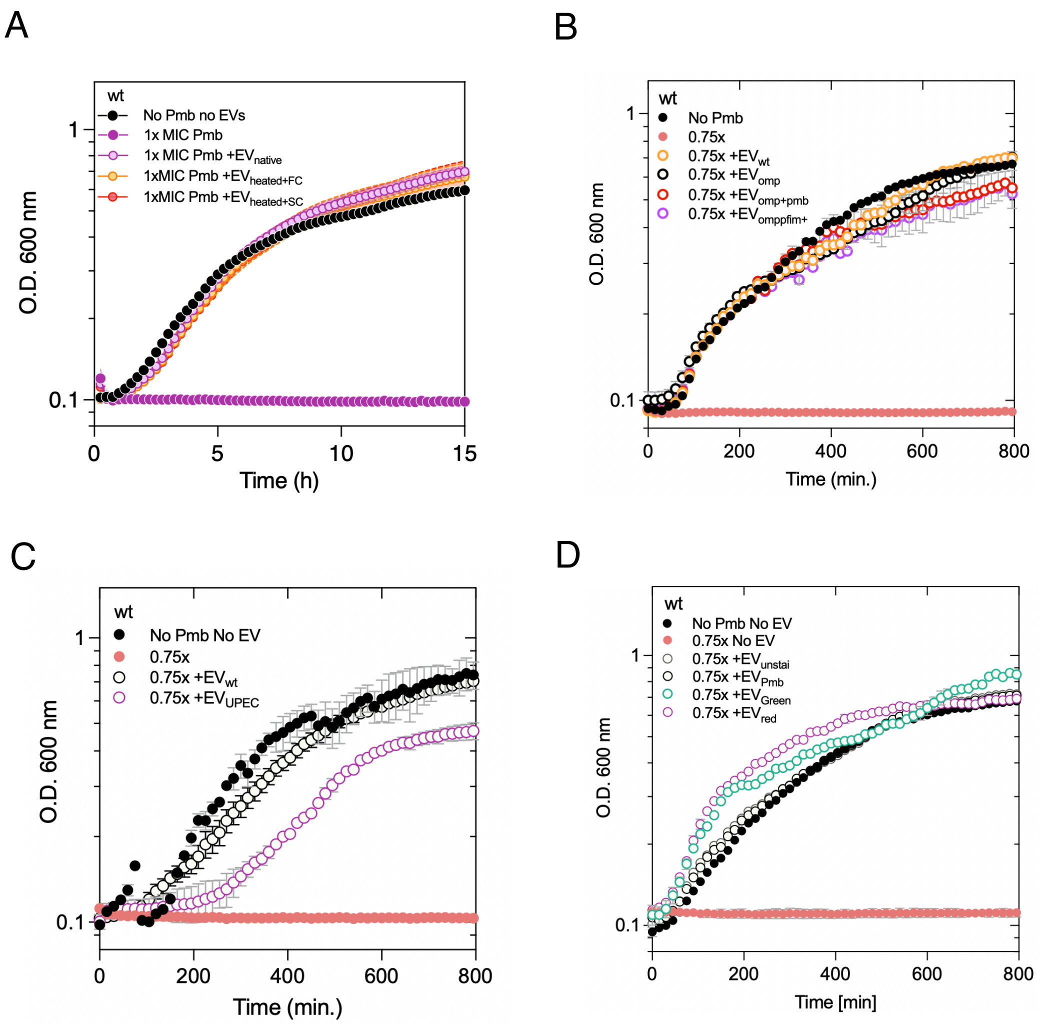

Fig. S5. Growth restoration effect and cell protection against Pmb antibiotic by heated EVs and EVs of various origins. (A) Effect of high temperature on EV protection role and growth recovery in the presence of Pmb (1x MIC). Purified EVs (from Δ*ompA* donor strain, 20h of growth)(concentration-normalized) were either untreated (placed at 4 °C immediately after purification), or heat-treated at 75 °C for 30 minutes and then allowed to cool down at room temperature (slow cool, SC) or heat-treated at 75 °C for 30 minutes and then placed immediately on ice then at 4 °C (fast cool, FC) prior to use. EVs and Pmb were added simultaneously at time 0, and growth was monitored by measuring OD600. **(B)** Effect of pure EVs (concentration normalized to 2.5 E+09 EV/ml) from different donor cells (wt, Δ*ompA*, Δ*ompA* +Pmb and hyper-fimbriated Δ*ompA* Δ*fim::Pclfim*$^{+}$*)* on cell growth in the presence of Pmb (0.75x MIC). **(C)** Effect of pure EVs from wt *E. coli* MG1655 and pathogenic *E.coli* UPEC, on cell growth under Pmb (0.75x MIC) conditions. **(D)** Effect of EVs (concentration normalized to 2.5 x10^{+9} EV/ml) stained with various fluorescent probes such as Fm1-43 (green), Fm4-64 (red), and Pmb$_{fl}$ which label EV membranes, on cell growth in the presence of Pmb (0.75x MIC). In all plots data are shown as mean ± SD (n=3).

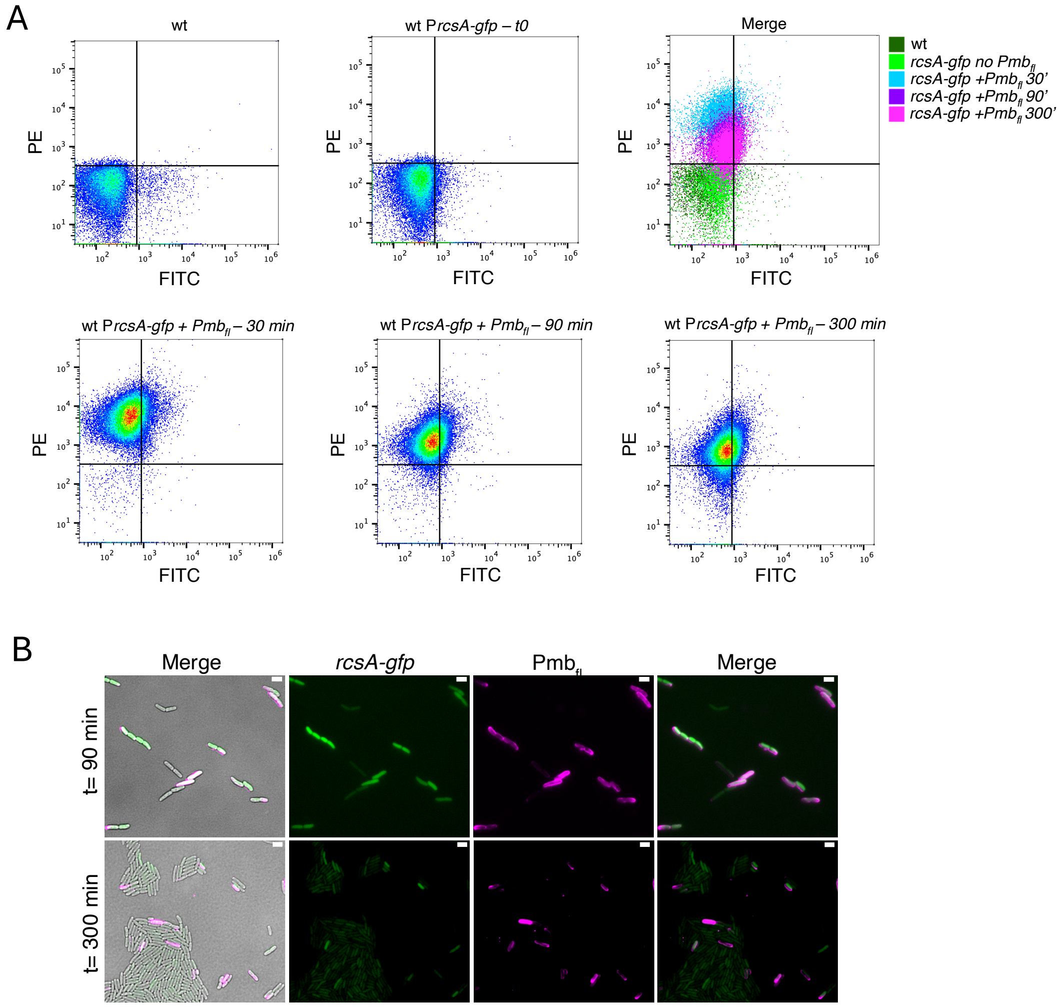

Fig. S6. Pmbfl insertion into cell membranes. (A) Representative pseudo-color plots displaying Pmbfl and *rcsA-gfp* fluorescence signals analyzed by flow cytometry. Subpopulations of live cells (wt or wt pPr*rcsA-gfp*) either exposed or not exposed to Pmbfl (0.5x MIC) for 30, 90 and 300 minutes, were identified and gated for membrane fluorescence analysis with a 560 nm laser (PE) and for envelop stress response analysis using a 488 nm laser (FITC). Merge pseudo-color plot is also shown. N=50,000 events were collected per sample. (**B**) Representative fluorescence microscopy images of *rcsA-gfp* expression following 90 and 300 minutes of exposure to Pmbfl (0.5x MIC). Merge of brightfield, FITC and TRITC channels are shown. Scale bar is 2μm.

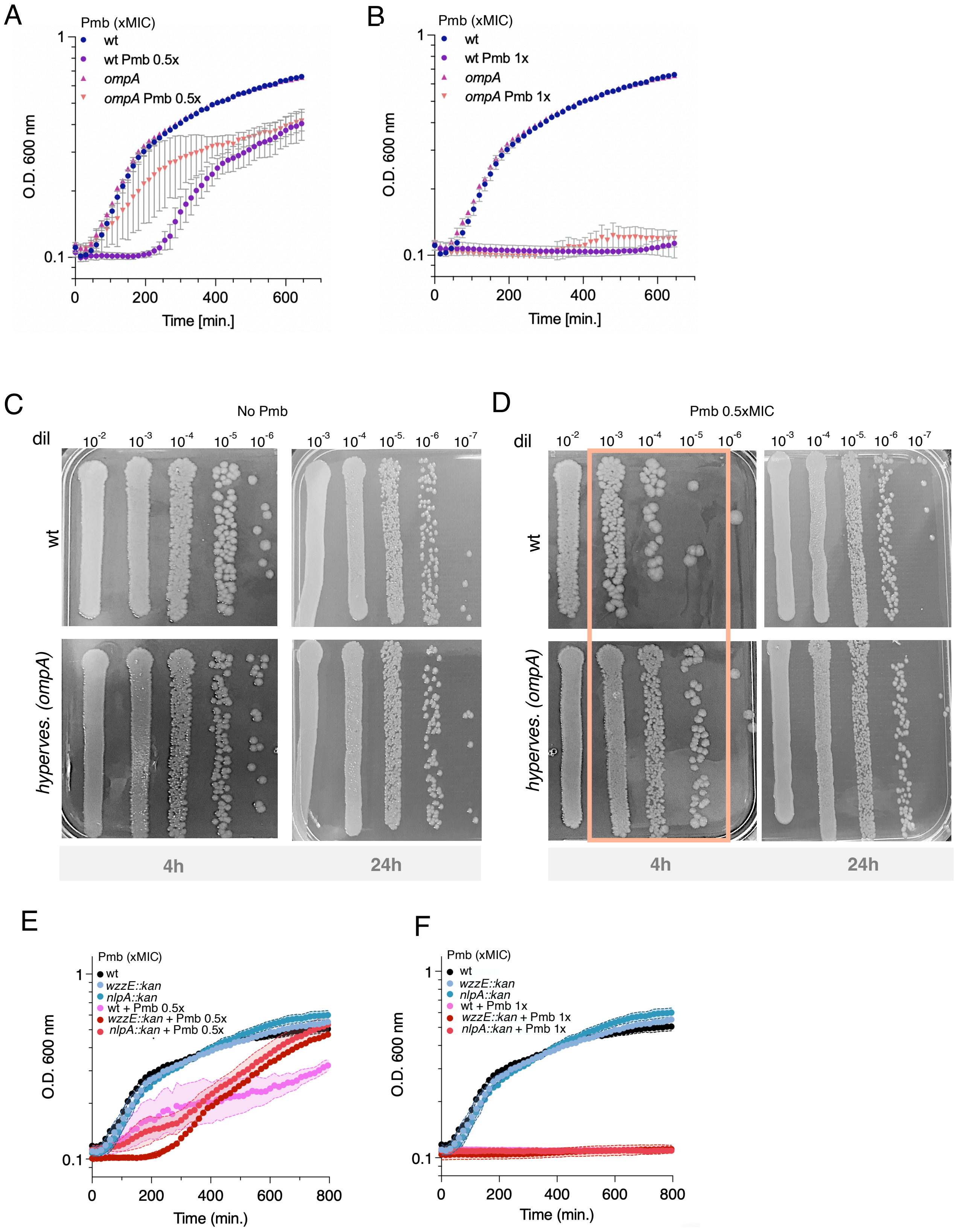

Fig. S7: Growth curves and survival of wt and D*ompA* strains in the presence or the absence of Pmb. (A-C) When cultured in plain LB (no Pmb), the wt and Δ*ompA* strains showed no significant differences in growth (A and B) or survival (C). The hyper-vesiculated Δ*ompA* strain exhibits a growth

(A) and survival **(D)** advantage (orange frame area) over the wt shortly after the addition of sub-MIC Pmb (0.5x MIC). However, this advantage is gone following prolonged treatment (24h) with sub-MIC Pmb (0.5x MIC) (D) or higher concentrations of Pmb (1x MIC) (B). Data are shown as mean ± SD (n=3). **(E-F)** Growth curves of wt (BW25113) and hypo-vesiculating mutant strains Δ*nlpA* and Δ*wzzE* (from KEIO collection) were measured in the absence or presence of Pmb (0.5x and 1x MIC). The lag phases of wt and Δ*nlpA* were comparable, with Δ*nlpA* displaying slightly slower growth, whereas Δ*wzzE* exhibited a prolonged lag phase (up to ~250 min), indicating delayed early adaptation to membrane stress in this mutant. Data are shown as mean and error range (n=2).

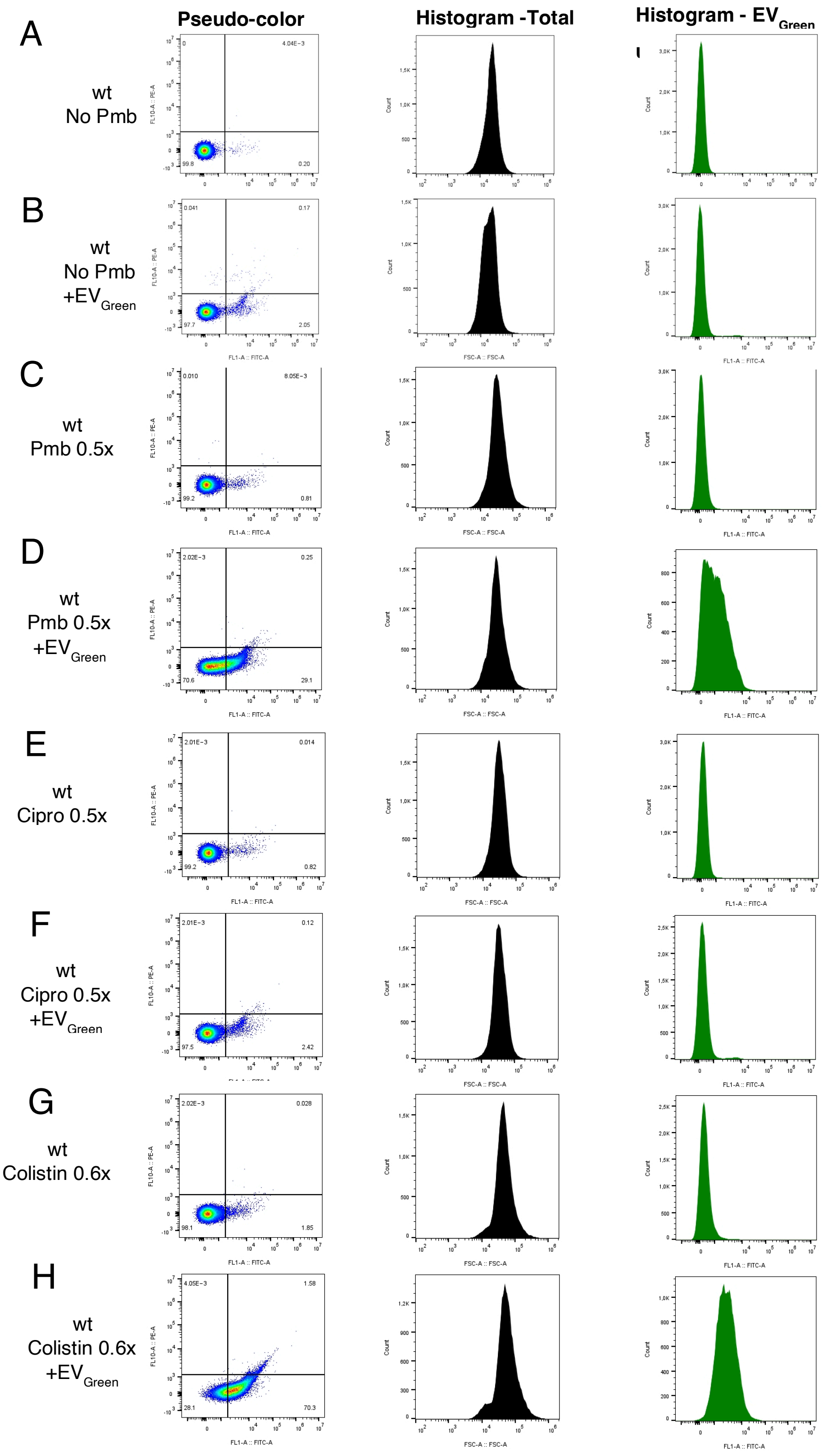

Fig. S8: EV uptake efficacy varies with the type of antibiotic. Histograms and pseudo-color plots showing EV uptake at cell membranes analysed by flow cytometry. Sub-populations of live cells (wt) were exposed to either no drug treatment **(A-B)** or treated with Pmb (0.5x MIC; 30 min) **(C-D)**, Ciprofloxacin (0.5x MIC; 30 min) **(E-F)**, Colistin (0.6x MIC; 30 min) **(G-H)**. After these treatments, pure EV_{Green} (concentration of ~$1x10^{+10}$ EVs; ~72 EVs/cell) were added for 10 min. Subpopulations were identified and gated for membrane fluorescence analysis using a 488 nm laser. Forward scatter (FSC) analysis provides information about the size of cells (total population). N=10,000 to 50,000 events were collected per sample.

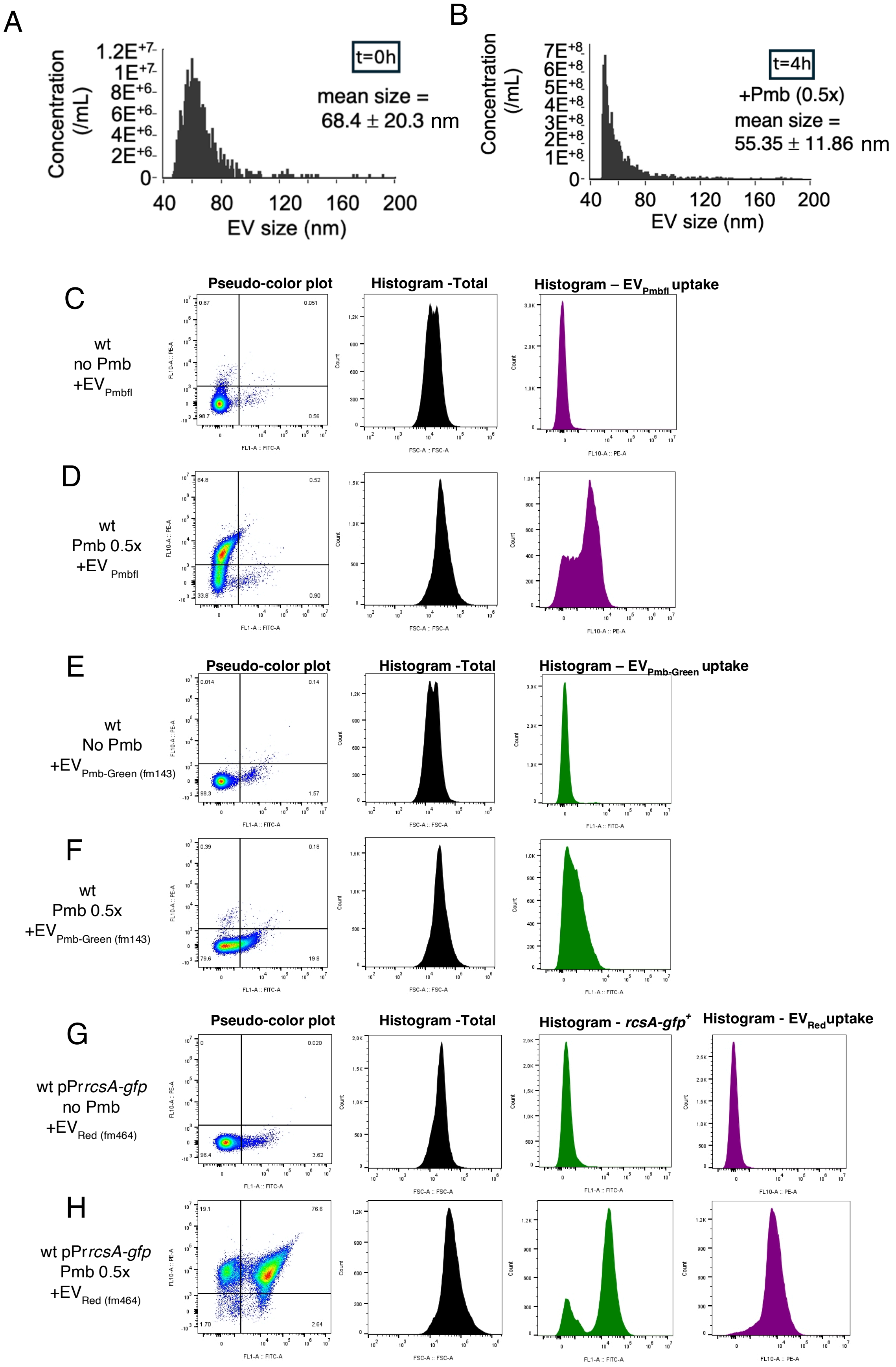

Fig. S9: EV uptake occurs with EVs of different origins and fluorescent probes. (A-B) Distribution plots of EV size and concentrations in the absence of Pmb (A) and in the presence of Pmb (0.5x MIC,

4h). Data were obtained at the nanoflow cytometer (Nanofcm). Mean size of EVs are indicated. **(C-H)** Histograms and pseudo-color plots of EV uptake signals at cell membranes analysed by flow cytometry. N=10,000 to 50,000 events were collected per sample. Subpopulations of live cells were exposed to either no drug treatment (C, E, G) or Pmb (0.5x MIC; 30 min) (D,F,H), followed by the addition of pure antibiotic-loaded EVs such as EV_{Pmbfl} (EVs purified from Δ*ompA* cells grown with Pmb_{fl} 0.16x MIC for 20 hours) (C and D) or $EV_{PmbGreen}$ (purified from Δ*ompA* cells grown with Pmb 0.25x MIC for 20 hours)(E and F) and regular EVs stained with Fm4-64 (EV_{Red}) (G and H). All EVs were added to a concentration of ~1x10^{+10} EVs; ~72 EVs/cell. These subpopulations were identified and gated for membrane fluorescence analysis using 488 nm or 560 nm lasers. Forward scatter (FSC) analysis provided information on cell size (total population).

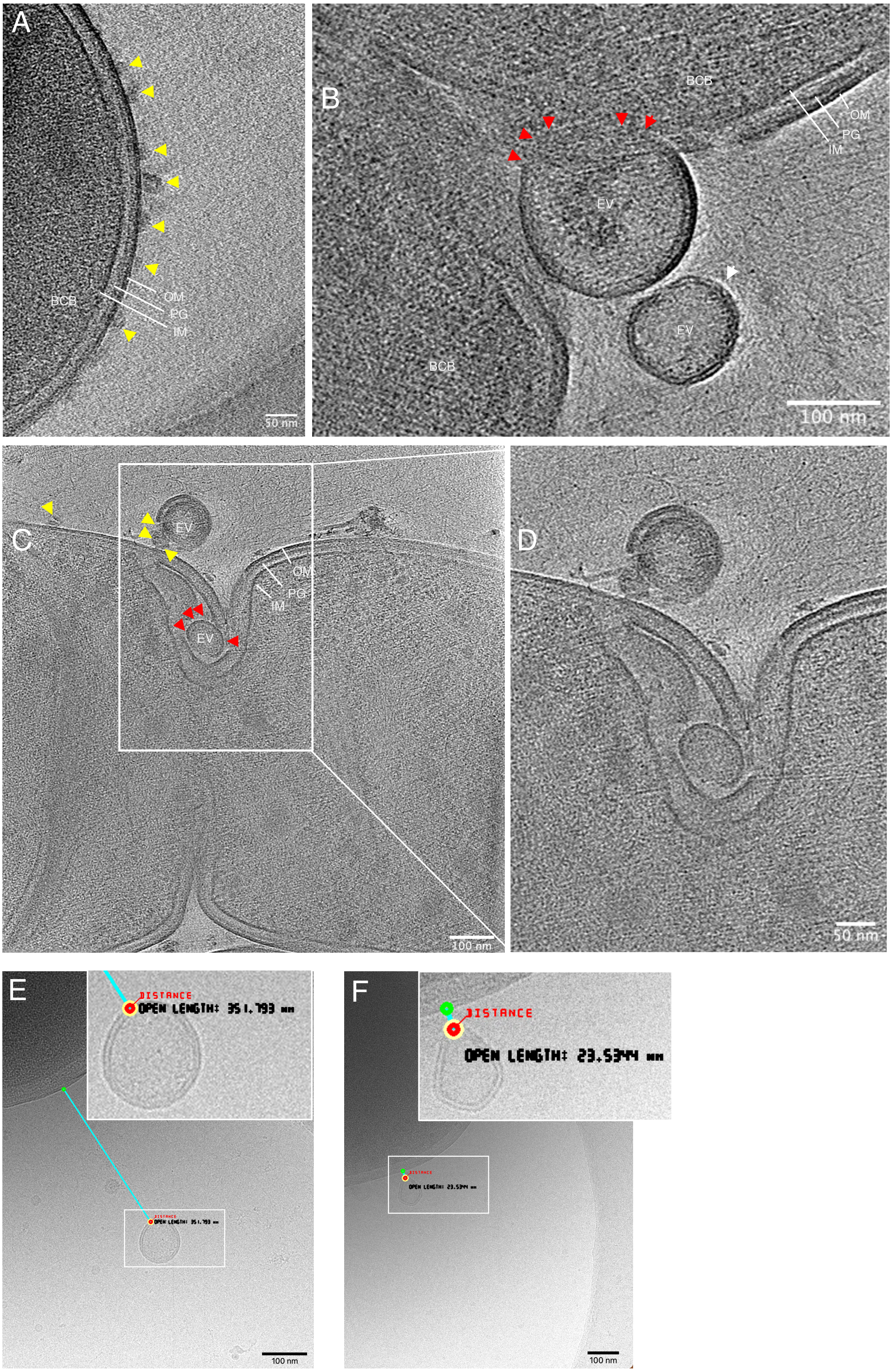

Fig. S10: Additional cryo-electron images (A-C) highlighting EV interactions at the membranes of *E. coli* cells challenged with Pmb (Refer to the Methods section in the main text for detailed methodology). Scale bar is indicated. The bacterial cell bodies (BCB) are indicated along with the outer

(OM) and inner (IM) membranes of the cell, the intermembrane peptidoglycan (PG) mesh, and the EVs in proximity to- (white arrowheads), adhering (yellow arrowheads) or fused (red arrowheads) to the outer membrane. Image in D is a blowup of selected area in C. **(E-F).** Representative example of snapshots of cryo-electron images used to measure the distances between cell membranes and EVs, in the absence (E) and in the presence (F) of Pmb, using IMOD software and reported in Fig. 5G. Inset panels show enlarged views of selected areas. Scale bars are indicated in all images.

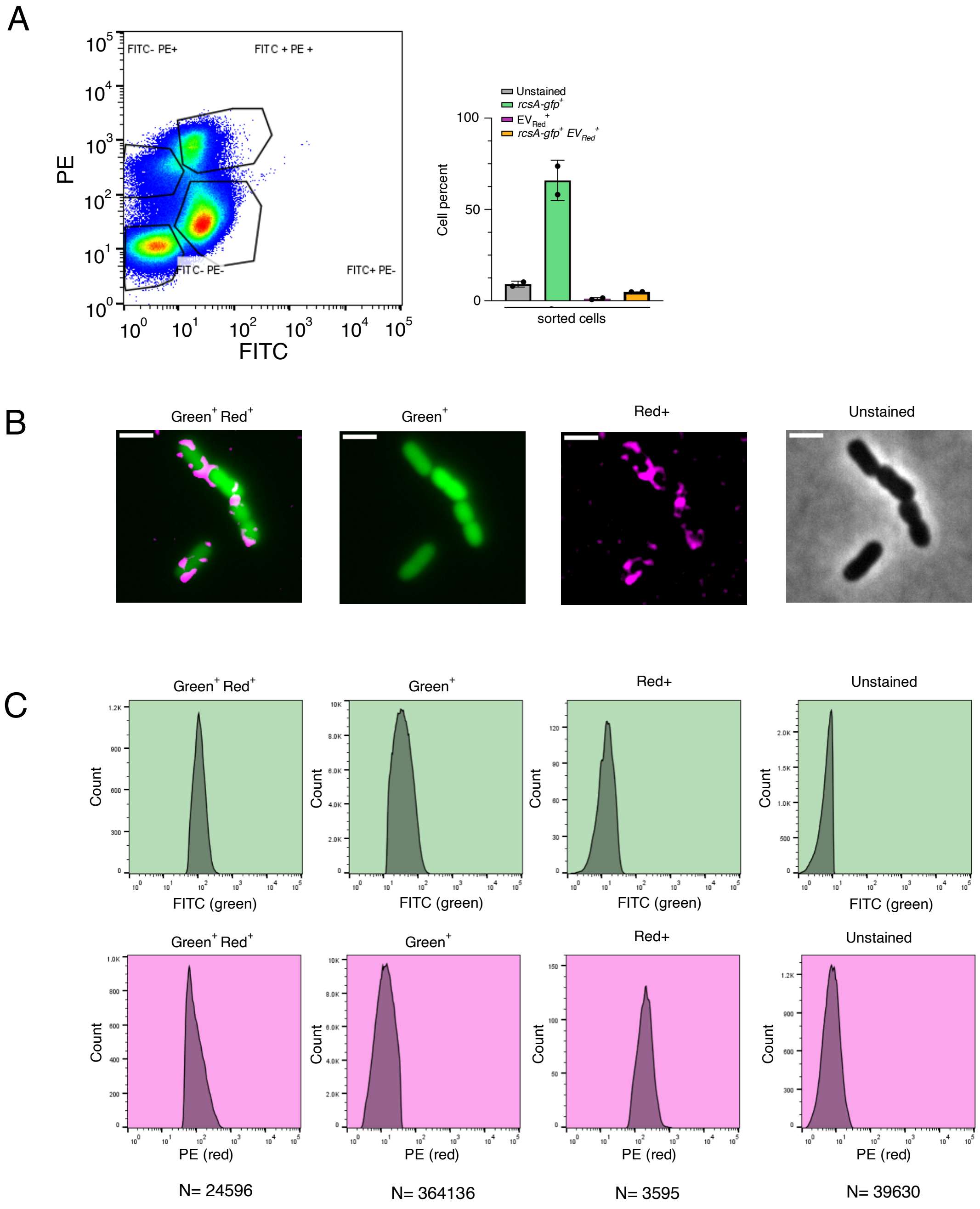

Fig. S11: Cell sorting of EV-patched cells for growth recovery. Wild-type pPr*rcsA-gfp* cells were grown for 30 minutes in the presence of Pmb at 0.5x MIC. Pure EV_{Red} was then added to a final concentration of ~ 1 x 10^{10} EVs (~72 EVs per cell) for 10 minutes (see Methods: "Sample preparation for EV uptake analysis"). **(A)** Gating strategy: Cells were sorted based on fluorescence intensity using the following gating criteria: FITC for *rcsA-gfp* positive cells ('stressed' cells), PE for EV_{Red}-positive cells ('patched' cells), FITC + PE for EV_{Red} + *rcsA-gfp* positive cells ('patched stressed' cells), and unstained (non-fluorescent) cells. Percentages of sorted subpopulations after gating are indicated (n=2). **(B)** Fluorescence and phase-contrast microscopy images of the gated subpopulations after sorting are provided. Scale bar: 2 µm. **(C)** The number of sorted cells (N) in each gate and corresponding histograms are indicated.

<u>**4 movies**</u>

Movie S1: Vesiculation in wild-type (wt) cells facilitates the clearance of Pmb_{fl}-damaged membranes. The scale bar represents 2 microns. Movie S1 was recorded over a 9-second duration [min:sec].

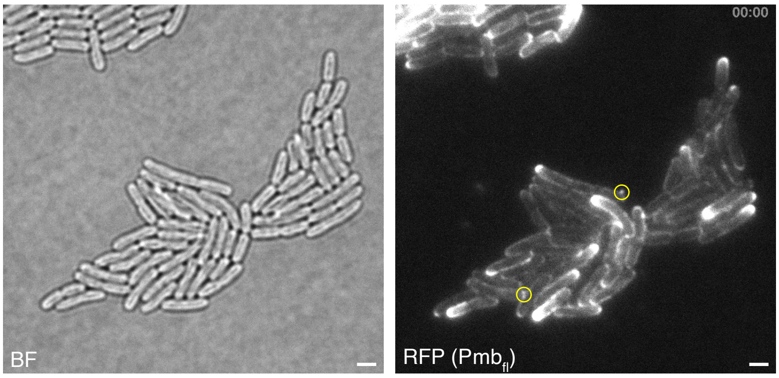

Movie S2 : Vesiculation in *ompA* cells promotes clearance of Pmb_{fl}-damaged. Scale bar is 2 microns. Movie S1 was recorded over a 9-second duration [min:sec]

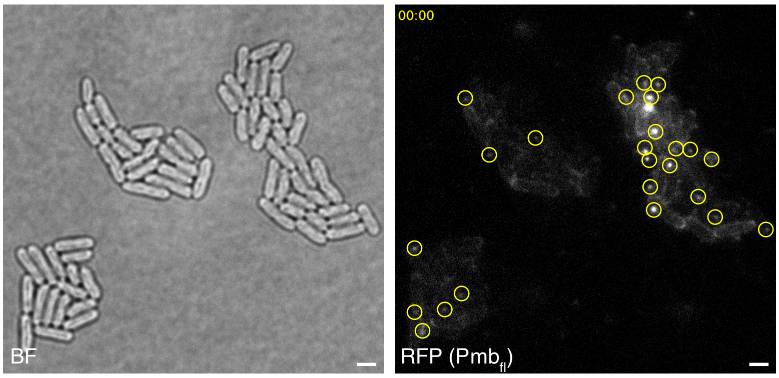

For both Movie S1 and Movie S2, Cells were grown on an LB agarose pad for 150 minutes following Pmb_{fl} treatment. Cell recovery and vesiculation were captured using fluorescence microscopy, with images taken in both Brightfield (left) and RFP (right) channels. EVs formed, released, or taken up by the bacteria are circled in yellow, with some EVs that are mobile and some that are not, as shown in our previous work (Bos et al. (*38*) DOI: 10.1126/sciadv.abd1033).

Movie 3: 3D Reconstructed tomogram of EV interaction with. *E. coli* cell membrane in the absence of Pmb treatment. (see Figure 4C for corresponding snapshot image). Scale bar is 50 nm.

Movie 4: 3D Reconstructed tomogram of EV interaction with. *E. coli* cell membrane in the absence of Pmb treatment. (see Figure 4D for corresponding snapshot image). Scale bar is 50 nm.

Full images of tomograms (movie S3 and S4) are accessible on Zenodo public repository 10.5281/zenodo.18075591 and full frames of movie S1 and S2 are accessible on Zenodo public repository 10.5281/zenodo.18076025
